# Electrochemical deposition as a universal route for fabricating single-atom catalysts

**DOI:** 10.1038/s41467-020-14917-6

**Published:** 2020-03-05

**Authors:** Zhirong Zhang, Chen Feng, Chunxiao Liu, Ming Zuo, Lang Qin, Xupeng Yan, Yulin Xing, Hongliang Li, Rui Si, Shiming Zhou, Jie Zeng

**Affiliations:** 1 Hefei National Laboratory for Physical Sciences at the Microscale, Key Laboratory of Strongly-Coupled Quantum Matter Physics of Chinese Academy of Sciences, Key Laboratory of Surface and Interface Chemistry and Energy Catalysis of Anhui Higher Education Institutes, Department of Chemical Physics, University of Science and Technology of China, 230026 Hefei, Anhui People’s Republic of China; 2 Shanghai Synchrotron Radiation Facility, Shanghai Institute of Applied Physics, Chinese Academy of Sciences, 201204 Shanghai, People’s Republic of China

**Keywords:** Catalyst synthesis, Electrocatalysis, Synthesis and processing

## Abstract

Single-atom catalysts (SACs) exhibit intriguing catalytic performance owing to their maximized atom utilizations and unique electronic structures. However, the reported strategies for synthesizing SACs generally have special requirements for either the anchored metals or the supports. Herein, we report a universal approach of electrochemical deposition that is applicable to a wide range of metals and supports for the fabrication of SACs. The depositions were conducted on both cathode and anode, where the different redox reactions endowed the SACs with distinct electronic states. The SACs from cathodic deposition exhibited high activities towards hydrogen evolution reaction, while those from anodic deposition were highly active towards oxygen evolution reaction. When cathodically- and anodically-deposited Ir single atoms on Co_0.8_Fe_0.2_Se_2_@Ni foam were integrated into a two-electrode cell for overall water splitting, a voltage of 1.39 V was required at 10 mA cm^−2^ in alkaline electrolyte.

## Abstract (web summary)

While single-atom catalysts exhibit intriguing catalytic performances and electronic structures, syntheses are often tailored to a particular system. Here, authors report electrochemical deposition as a universal approach for the fabrication of single-atom catalysts over range of metals and supports.

## Introduction

Single-atom catalysts (SACs), with uniform isolated metal atoms anchored on supports, have received wide interests in recent years due to their extraordinary catalytic performance towards various reactions such as water splitting, oxygen reduction, CO_2_ hydrogenation, and methane conversion^1–4^. As the supported metals are downsized to the minimum, SACs reach the utmost atom-utilization efficiency. Moreover, the atomically dispersed metal species usually exhibit unique electronic states due to distinctive coordinated environments, strong metal-support interactions, and quantum size effects^5–7^. In such catalysts, the identical geometric structure of active centers also offers an ideal platform for understanding catalytic mechanisms at the atomic-scale level^8,9^.

SACs are not readily available since the individual metal atoms incline to diffuse and aggregate into clusters during the synthesis in order to lower surface energy. The last decade has witnessed the development of strategies for fabricating SACs. These strategies include chemical vapor deposition^10^, atomic layer deposition^11^, pyrolysis^12^, wet chemistry methods^13^, photochemical methods^14^, and atom trapping^15^. However, these strategies usually have special requirements for either the anchored metals or the supports. For instance, the pyrolysis method generally yielded carbon-based supports because of the decomposition of metal-organic complexes^16,17^. Moreover, wet chemistry methods are commonly a trial-and-error process, since no consensus has been reached on the synthetic mechanism^18,19^. The atom trapping not only required the mobile metal species, but also demanded trapping sites on the supports^15,20^. Therefore, it is highly desired but challenging to develop a universal approach that is applicable to a wide range of metals and supports for the fabrication of SACs.

Here, we report electrochemical deposition as a general strategy to produce SACs. More than 30 different SACs are successfully obtained from cathodic or anodic deposition just by varying metal precursors and supports. Intriguingly, the cathodically and anodically deposited single atoms for the same metal exhibit different electronic states. Moreover, electrocatalytic tests demonstrat that these SACs obtained from cathodic deposition show a great potential for catalyzing hydrogen evolution reaction, while those from anodic deposition are promising candidates for oxygen evolution catalysts. Specially, Ir single atoms on Co_0.8_Fe_0.2_Se_2_ nanosheets from the cathodic deposition require only an overpotential of 8 mV to reach a current density of 10 mA cm^−2^ for alkaline hydrogen evolution reaction. Furthermore, a two-electrode cell assembled by cathodically and anodically deposited Ir single atoms on Co_0.8_Fe_0.2_Se_2_@Ni foam exhibits a voltage of 1.39 V at 10 mA cm^−2^ for overall water splitting in alkaline electrolyte.

## Results

### Synthesis and characterizations of single-atom catalysts

**Fig. 1:**
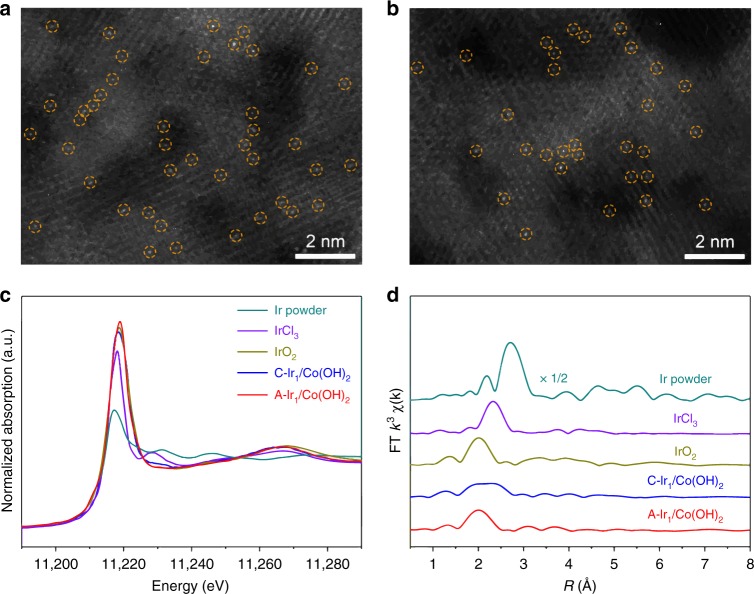
Structural characterizations of Ir single atoms on Co(OH)_2_ nanosheets. **a**, **b** HAADF-STEM images of C-Ir_1_/Co(OH)_2_ (**a**) and A-Ir_1_/Co(OH)_2_ (**b**). Singly dispersed Ir atoms are indicated by yellow circles. **c**, **d** Normalized XANES (**c**) and EXAFS (**d**) spectra at the Ir *L*_3_-edge for C-Ir_1_/Co(OH)_2_ and A-Ir_1_/Co(OH)_2_. Ir powder, IrCl_3_, and IrO_2_ were used as references.

To demonstrate the synthetic procedure, we take the deposition of Ir single atoms on Co(OH)_2_ nanosheets as an example. Specially, we used a standard three-electrode system, where Co(OH)_2_ nanosheets were loaded onto a glassy carbon electrode as the working electrode (Supplementary Fig. 1). Soluble metallic salts with a low concentration, 100 μM IrCl_4_ in this case, were added into the 1 M KOH electrolyte as the metal precursors. The depositing potential was set from 0.10 to −0.40 V in cathodic deposition and from 1.10 to 1.80 V in anodic deposition for one scanning cycle, with a scanning rate of 5 mV s^−1^. The scanning cycle was repeated for ten times to obtain C-Ir_1_/Co(OH)_2_ from the cathode and three times to obtain A-Ir_1_/Co(OH)_2_ from the anode. Transmission electron microscopy (TEM) images and X-ray diffraction (XRD) patterns show the absence of Ir-based clusters or nanoparticles in the as-deposited samples (Supplementary Fig. 2). The energy-dispersive X-ray elemental mapping revealed that Ir species of both samples were homogeneously dispersed on the substrates (Supplementary Fig. 3). The aberration-corrected high-angle annular dark-field scanning TEM (HAADF-STEM) images of C-Ir_1_/Co(OH)_2_ and A-Ir_1_/Co(OH)_2_ further demonstrated that isolated Ir atoms were uniformly distributed on the substrates (Fig. 1, a, b). The mass loadings of Ir in C-Ir_1_/Co(OH)_2_ and A-Ir_1_/Co(OH)_2_ were determined to be 2.0 and 1.2 wt%, respectively, by inductively coupled plasma atomic emission spectroscopy (ICP-AES) (Supplementary Table 1).

To determine the electronic structures and coordination environment of Ir atoms in the as-deposited SACs, we performed X-ray absorption near-edge spectroscopy (XANES) and extended X-ray absorption fine structure (EXAFS) measurements. Figure 1c shows the Ir *L*_3_-edge XANES spectra of C-Ir_1_/Co(OH)_2_ and A-Ir_1_/Co(OH)_2_. The white line intensity for C-Ir_1_/Co(OH)_2_ lay between that for IrCl_3_ and IrO_2_, indicating that the oxidation state of Ir was between +3 and +4. By comparison, the white line intensity for A-Ir_1_/Co(OH)_2_ was higher than that for IrO_2_, indicating a higher oxidation state of Ir than +4. The oxidation states of Ir in both samples were also confirmed by X-ray photoelectron spectroscopy (XPS) (Supplementary Fig. 4). As shown in the EXAFS spectra of C-Ir_1_/Co(OH)_2_, two peaks appeared at 1.97 Å and 2.32 Å, which were contributed to the first coordination shell of Ir-O and Ir-Cl, respectively (Fig. 1d). The fitting results indicated a coordination number (*CN*) of 3.3 for Ir-O contribution and 3.1 for Ir-Cl contribution (Supplementary Fig. 5 and Supplementary Table 2). No other typical peaks for Ir-Ir contribution at 2.71 Å were observed, revealing the atomic dispersion of Ir atoms throughout the whole C-Ir_1_/Co(OH)_2_. In the EXAFS spectra of A-Ir_1_/Co(OH)_2_, only one prominent peak showed at 2.01 Å for Ir-O contribution (Fig. 1d). The fitting results indicated a *CN* of 5.8 for Ir-O contribution (Supplementary Fig. 5 and Supplementary Table 2). The absence of peaks for Ir-Ir contribution also confirmed the atomically dispersed Ir in A-Ir_1_/Co(OH)_2_.

### Synthetic mechanism

**Fig. 2:**
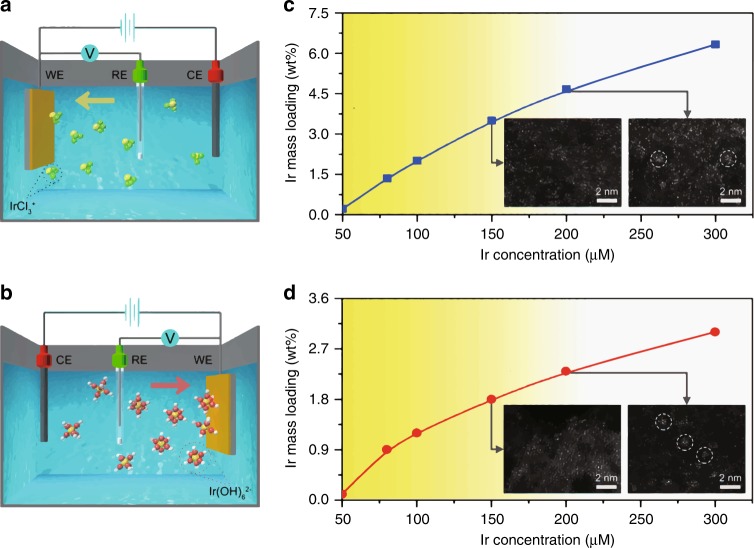
Electrochemical deposition mechanism. **a**, **b** Schematic of cathodic (**a**) and anodic (**b**) deposition of Ir species. The yellow, green, red, and white spheres represent Ir, Cl, O, and H atoms, respectively. A standard three-electrode system was used, with Co(OH)_2_ nanosheets loaded on a glassy carbon electrode as the working electrode (WE), a carbon rod as the counter electrode (CE), and an Ag/AgCl electrode as the reference electrode (RE). **c**, **d** Ir mass loadings as a function of Ir concentration in the 1 M KOH electrolyte for cathodic (**c**) and anodic (**d**) deposition. The scanning cycle number was kept at ten for cathodic deposition and three for anodic deposition. The color gradient from yellow to white indicates the transition from single atoms to clusters with increasing Ir concentration. The inset images correspond to the HAADF-STEM images of the samples obtained at a certain concentration. The areas in white circles indicate the agglomerations of single atoms to form clusters.

We propose a typical schematic for cathodic and anodic deposition of Ir species in KOH electrolyte according to the above results. For the cathodic deposition, iridium cations were driven towards the cathode by the applied electric field and deposited on the supports (Fig. 2a). Based on the *CN* of 3.1 for Ir-Cl contribution, we suppose that the cations were IrCl_3_^+^. According to the *CN* of 3.3 for Ir-O contribution, IrCl_3_^+^ cations were proposed to coordinate with three O atoms on Co(OH)_2_. Moreover, the deposition of IrCl_3_^+^ was accompanied with a reduction of Ir ions under the negative electronic field, giving rise to the oxidation state of Ir in C-Ir_1_/Co(OH)_2_ lower than +4. For the anodic deposition, iridium anions are driven by the electric field towards the anode (Fig. 2b). The iridium anions were likely derived from the combination of Ir^4+^ in the precursors with OH^−^ in the electrolyte. Ultraviolet–Visible (UV) adsorption spectrum of the 1 M KOH electrolyte containing 100 μM IrCl_4_ showed a prominent peak at 318 cm^−1^, corresponding to the adsorption of Ir(OH)_6_^2−^ (Supplementary Fig. 6)^21^. This suggested that Ir(OH)_6_^2−^ anions act as the depositing species for anodic deposition, which is in accordance with the EXAFS result of A-Ir_1_/Co(OH)_2_ with a *CN* of 5.8 from Ir-O contribution. Meanwhile, this anodic deposition was coupled with an oxidation process of the anions, resulting in the oxidation state of Ir in A-Ir_1_/Co(OH)_2_ higher than +4.

To get an insight into the deposition mechanism of SACs, we further varied the concentration of Ir precursors, the number of scanning cycles, and the scanning rate. Cathodic depositions were conducted in 1 M KOH electrolytes via changing the Ir concentration from 50 to 300 μM for ten scanning cycles. As shown in Fig. 2c, the mass loadings of Ir species constantly increased with the growing Ir concentration. When the Ir concentration was increased to 150 μM, Ir single atoms were still obtained at a mass loading of 3.5%. When the concentration was further raised to 200 μM, Ir clusters appeared at a mass loading of 4.7%. As such, for the formation of SACs, there exists an upper limit of mass loadings between 3.5% and 4.7%. When we changed the scanning cycles at the Ir concentration of 100 μM, the upper limit of mass loadings lay between 3.3% and 3.9% for the formation of SACs (Supplementary Fig. 7a). Moreover, the Ir mass loadings increased with decreasing scanning rate, where the upper limit of mass loadings lay between 2.6% and 3.6% for the formation of SACs (Supplementary Fig. 8a). This upper limit was roughly consistent with those obtained via varying the Ir concentration and the scanning cycles. For the anodic depositions, similar phenomena were also observed, whenever the Ir concentration, the scanning cycle, and the scanning rate were changed (Fig. 2c and Supplementary Figs. 7b and 8b). These electrochemical deposition processes resemble the molecular mechanism of nucleation in the solution-phase synthesis, where the support can be regarded as the solvent^22^. In this case, the upper limit of mass loading for SACs corresponds to the level of minimum supersaturation on the support. When the mass loadings of metal on the support exceeds the minimum supersaturation level, singly dispersed metal atoms starts to nucleate into clusters. Therefore, the formation of SACs strongly relies on controlling the mass loading below the level of minimum supersaturation. Moreover, since the isolated single atoms are usually anchored to the support through defects or strong metal-support interaction, the value of minimum supersaturation might be relevant to the number of depositing sites such as vacancies, edges, or steps.

**Fig. 3:**
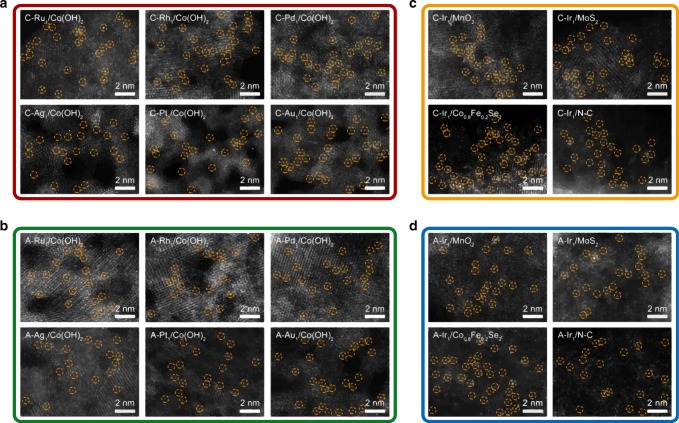
Generality of electrochemical deposition for synthesizing SACs. **a**, **b** Cathodically (**a**) and anodically (**b**) deposited metal single atoms on Co(OH)_2_ nanosheets (denoted as C-M_1_/Co(OH)_2_ and A-M_1_/Co(OH)_2_, M = Ru, Rh, Pd, Ag, Pt, and Au). **c**, **d** Cathodically (**c**) and anodically (**d**) deposited Ir single atoms on different supports (denoted as C-Ir_1_/support and A-Ir_1_/support, support = MnO_2_, MoS_2_, Co_0.8_Fe_0.2_Se_2_, and N-C). The depositions were all conducted in a 1 M KOH electrolyte containing 100 μM metal precursors. For cathodic deposition, the deposition process was conducted in a potential range from 0.10 to −0.40 V for ten scanning cycles. For anodic deposition, the deposition process was conducted in a potential range from 1.10 to 1.80 V for three scanning cycles. The singly dispersed metal atoms are marked by yellow circles.

To test the generality of this method for synthesizing SACs, we replaced the Ir precursor with other precursors of 4*d* and 5*d* metals, including Ru, Rh, Pd, Ag, Pt, and Au. HAADF-STEM images illustrated that all these metal species were successfully deposited and atomically dispersed on Co(OH)_2_ nanosheets under similar depositing conditions (Fig. 3, a, b). The atomic dispersion of metal species was further supported by in-situ diffuse reflectance infrared Fourier transform (DRIFT) experiments using CO as a probe molecule (Supplementary Fig. 9). Besides, the cathodic and anodic deposition of the same precursor resulted in different oxidation states of the deposited species (Supplementary Fig. 10). To further extend the applicability of this method, we utilized the same depositing method and obtained SACs comprising of other 3*d* and 4*d* transitional metals on nitrogen-doped carbon (N-C) support as well (Supplementary Fig. 11). We also deposited Ir single atoms onto other supports following the same procedure of synthesizing Ir_1_/Co(OH)_2_. These supports include MnO_2_ nanosheets, MoS_2_ nanosheets, Co_0.8_Fe_0.2_Se_2_ nanosheets, and N-C (Supplementary Fig. 12). Likewise, the Ir single atoms were successfully deposited on these supports through the cathodic or anodic deposition (Fig. 3, c, d). The absence of metal-metal bond in the EXAFS spectra further confirmed the atomic dispersion of these single atoms, in good agreement with the HAADF-STEM results (Supplementary Fig. 13). To be noted, due to the applied oxidative potential, the surface of these substrates underwent reconstructions during the anodic deposition process. The Co(OH)_2_, MnO_2_, MoS_2_, and Co_0.8_Fe_0.2_Se_2_ substrate was partially transferred to oxyhydroxides, amorphous phase, oxides, and oxyhydroxides, respectively, leading to vague lattice fringes in the HAADF-STEM images (Supplementary Fig. 2d and Supplementary Fig. 14). In addition, we have also applied this deposition method in acid electrolyte. Ir single atoms were successfully obtained from both cathodic and anodic deposition in 0.5 M H_2_SO_4_ (Supplementary Fig. 15). Therefore, the electrochemical deposition is a universal method that is applicable to a wide range of metals and supports for the fabrication of SACs.

### Catalytic properties towards water splitting

**Fig. 4:**
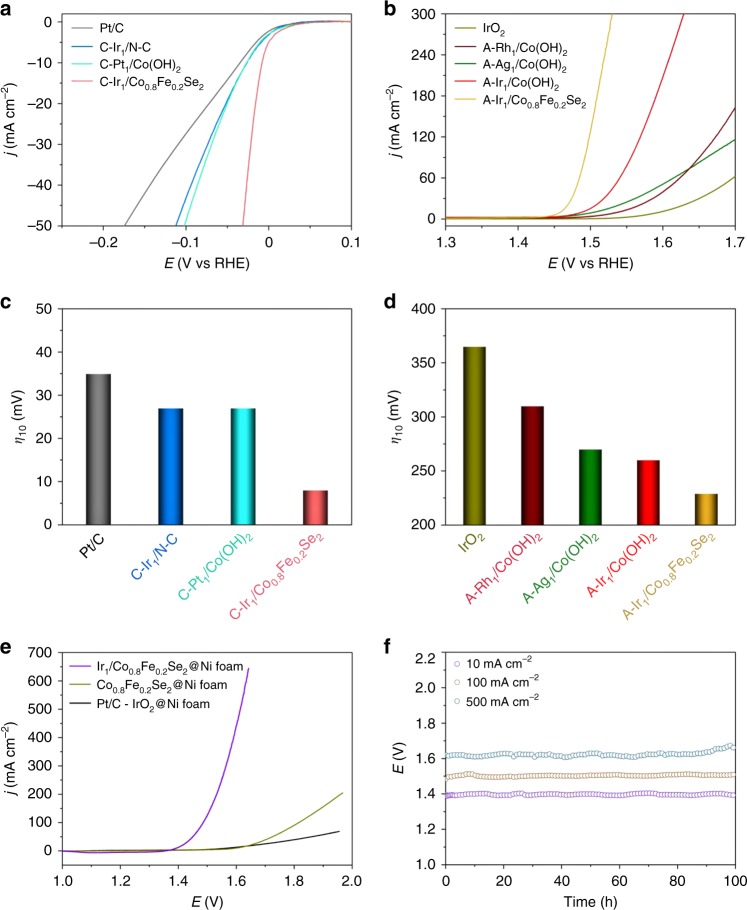
Electrocatalytic performances of SACs for water splitting. **a**, **b** Polarization curves of cathodically deposited SACs for HER (**a**) and anodically deposited SACs for OER (**b**). The measurements were all conducted in 1 M KOH. **c**, **d** The overpotentials at 10 mA cm^−2^ for HER (**c**) and OER (**d**). The commercial Pt/C and IrO_2_ were used as references for HER and OER, respectively. **e** Polarization curves of Ir_1_/Co_0.8_Fe_0.2_Se_2_@Ni foam for overall water splitting in the two-electrode cell. The commercial Pt/C-IrO_2_ coupled electrolyzer was used as a reference. **f** Chronopotentiometric curves of Ir_1_/Co_0.8_Fe_0.2_Se_2_@Ni foam for overall water splitting at 10, 100, and 500 mA cm^−2^ for 100 h.

Based on the library of obtained SACs, we searched for efficient catalysts towards water splitting, one of the most promising reactions for renewable hydrogen production. This chemical process consists of two half-reactions, including hydrogen evolution (HER) and oxygen evolution (OER) reactions^23,24^. The polarization curves of cathodically deposited SACs for HER and anodically deposited SACs for OER were recorded in 1 M KOH electrolyte (Fig. 4, a, b and Supplementary Fig. 16). It is worth noting that C-Ir_1_/N-C, C-Pt_1_/Co(OH)_2_, and C-Ir_1_/Co_0.8_Fe_0.2_Se_2_ exhibited higher activities towards HER relative to the commercial Pt/C (Fig. 4c). Strikingly, C-Ir_1_/Co_0.8_Fe_0.2_Se_2_ delivered an overpotential of 8 mV at a current density of 10 mA cm^−2^, comparable to the state-of-the-art HER catalysts in alkaline media (Supplementary Table 3). Towards OER, A-Rh_1_/Co(OH)_2_, A-Ag_1_/Co(OH)_2_, A-Ir_1_/Co(OH)_2_, and A-Ir_1_/Co_0.8_Fe_0.2_Se_2_, were found to be highly active compared with the benchmark IrO_2_. Specially, A-Ir_1_/Co_0.8_Fe_0.2_Se_2_ only needed an overpotential as low as 230 mV to reach 10 mA cm^−2^, which was 135 mV lower than that of IrO_2_ (Fig. 4d). The prominent HER and OER activity of Ir single atoms on Co_0.8_Fe_0.2_Se_2_ might originate from highly efficient charge transfer ability of the support and strong metal-support interaction (Supplementary Fig. 17)^25–28^.

Inspired by the high activities of as-deposited SACs towards HER and OER, we assembled C-Ir_1_/Co_0.8_Fe_0.2_Se_2_ and A-Ir_1_/Co_0.8_Fe_0.2_Se_2_ into a two-electrode cell for overall water splitting. The Ir_1_/Co_0.8_Fe_0.2_Se_2_ electrolyzer only needed a cell voltage of 1.48 V to reach a current density of 10 mA cm^−2^ and maintained the performance over 30 h (Supplementary Fig. 18). In order to improve the overall water splitting activity, the Co_0.8_Fe_0.2_Se_2_ nanosheets were further in-situ grown on Ni foam (denoted as Co_0.8_Fe_0.2_Se_2_@Ni foam) to replace the glassy carbon electrode for more catalyst loading, higher conductivity, and better adherence of the catalysts (Supplementary Fig. 19)^29^. Consequently, the overpotential needed to reach 10 mA cm^−2^ was further decreased to 4 mV for C-Ir_1_/Co_0.8_Fe_0.2_Se_2_@Ni foam during HER, and to 140 mV for A-Ir_1_/Co_0.8_Fe_0.2_Se_2_@Ni foam during OER, respectively (Supplementary Fig. 20, a, b). Afterwards, C-Ir_1_/Co_0.8_Fe_0.2_Se_2_@Ni foam and A-Ir_1_/Co_0.8_Fe_0.2_Se_2_@Ni foam were assembled into a two-electrode cell (denoted as Ir_1_/Co_0.8_Fe_0.2_Se_2_@Ni foam) for overall water spiltting (Supplementary Fig. 21a). Figure 4e shows the polarization curves for the Ir_1_/Co_0.8_Fe_0.2_Se_2_@Ni foam electrolyzer in comparison with the Pt/C-IrO_2_ coupled electrolyzer. For Ir_1_/Co_0.8_Fe_0.2_Se_2_@Ni foam, only a cell voltage of 1.39 V was required to reach a current density of 10 mA cm^−2^, substantially lower than that (1.57 V) for the Pt/C-IrO_2_ electrolyzer (Supplementary Fig. 21b). Moreover, the Faradaic efficiency of the two-electrode cell was estimated to be >99% towards both HER and OER, indicating that the current density only derived from water splitting (Supplementary Fig. 21, c, d). Notably, Ir_1_/Co_0.8_Fe_0.2_Se_2_@Ni foam readily drove the cell to a current density as high as 500 mA cm^−2^ at a voltage of only 1.62 V, which benefits for practical application in industry^30^. To the best of our knowledge, Ir_1_/Co_0.8_Fe_0.2_Se_2_@Ni foam outperformed all the reported electrocatalysts for overall water splitting in alkaline electrolyte up to date (Supplementary Table 4). Furthermore, Ir_1_/Co_0.8_Fe_0.2_Se_2_@Ni foam electrolyzer maintained its performance for over 100 h at a wide range of current densities in the long-term durability tests (Fig. 4f). Structural characterizations showed that Ir_1_/Co_0.8_Fe_0.2_Se_2_@Ni foam showed no obvious change after the durability tests (Supplementary Fig. 22). The excellent stability of Ir_1_/Co_0.8_Fe_0.2_Se_2_@Ni foam electrolyzer should be ascribed to the high stability of the Co_0.8_Fe_0.2_Se_2_@Ni foam substrate (Supplementary Fig. 20, c, d) and the unique deposition strategy under the same conditions as for electrochemical measurements.

## Discussion

In conclusion, we develop electrochemical deposition as a universal approach applicable to a wide range of metals and supports for the fabrication of SACs. The SACs from cathodic deposition exhibited high activities towards hydrogen evolution reaction, while those from anodic deposition were highly active towards oxygen evolution reaction. When cathodically- and anodically deposited Ir single atoms on Co_0.8_Fe_0.2_Se_2_@Ni foam were integrated into a two-electrode cell for overall water splitting, a record-low voltage of 1.39 V was required at 10 mA cm^−2^ in alkaline electrolyte. The highly active and stable Ir_1_/Co_0.8_Fe_0.2_Se_2_@Ni foam holds the promises for large-scale hydrogen generation. Our method to synthesize SACs provides a general route to exploit highly efficient catalysts for targeted chemical reactions.

## Methods

### Chemicals and materials

Iridium (IV) chloride hydrate (IrCl_4_·xH_2_O), sodium tetrachloropalladate(II) (Na_2_PdCl_4_), and gold (III) chloride hydrate (HAuCl_4_·xH_2_O) were purchased from Aladdin. Sodium hexachlororhodate (III) dodecahydrate (Na_3_RhCl_6_·12H_2_O) was purchased from Alfa Aesar. Commercial Pt/C catalyst (20 wt%, Pt nanoparticles with the size ranging from 2 to 5 nm) was purchased from Johnson Matthey Corporation. Potassium hydroxide (KOH), sodium chloride (NaCl), hexamethylenetetramine, 2-methylimidazole, hexaammonium heptamolybdate tetrahydrate ((NH_4_)_6_Mo_7_O_24_·4H_2_O), ethanol, methanol, thiourea, ethanolamine, sodium hydroxide (NaOH), selenium powder (Se), hydrazine, dimethylformamide (DMF), vanadium (III) chloride (VCl_3_), chromium (III) chloride (CrCl_3_), manganese (II) chloride tetrahydrate (MnCl_2_·4H_2_O), ferric (III) chloride (FeCl_3_), cobalt (II) chloride hexahydrate (CoCl_2_·6H_2_O), nickel (II) chloride (NiCl_2_), copper (II) chloride (CuCl_2_), zinc (II) nitrate hexahydrate (Zn(NO_3_)_2_·6H_2_O), ruthenium (III) chloride (RuCl_3_), silver nitrate (AgNO_3_), chloroplatinic (II) acid (H_2_PtCl_4_), and iridium (IV) oxide (IrO_2_) were purchased from Shanghai Chemical Reagent Company. All the other chemicals were of analytical grade and used without further purification. All aqueous solutions were prepared using deionized water with a resistivity of 18.2 MΩ cm^−1^.

### Synthesis of Co(OH)_2_ nanosheets

Co(OH)_2_ nanosheets were synthesized using a modified co-precipitation method^31^. In a typical procedure, CoCl_2_·6H_2_O (2 mmol), NaCl (10 mmol), and hexamethylenetetramine (12 mmol) were dissolved in a mixture of deionized water (200 mL) and ethanol (20 mL). The solution was magnetically stirred at 90 ^o^C for 2 h, and cooled down to room temperature. The as-obtained products were washed three times with deionized water and ethanol. The final products were dried at 40 ^o^C under vacuum overnight.

### Synthesis of N-C

The synthesis of N-C was based on a previously reported method^32^. Typically, Zn(NO_3_)_2_·6H_2_O (1.68 g) was dissolved in 80 mL of methanol. A mixture of 2-methylimidazole (3.70 g) and methanol (80 mL) was added into the above solution under vigorous stirring for 24 h. The products were separated by centrifugation and washed three times with methanol. Then the as-obtained products were dried at 50 ^o^C under vacuum overnight. Afterwards, the dried powder was further activated at 200 ^o^C under vacuum for 24 h. Then 500 mg of activated powder was heated to 900 ^o^C with a heating rate of 5 ^o^C  min^−1^ and carbonized at 900 ^o^C under N_2_ atmosphere for 2 h. After naturally cooling down to room temperature, nitrogen-doped porous carbon was obtained.

### Synthesis of MnO_2_ nanosheets

In the synthesis of MnO_2_ nanosheets, 0.04 g of MnCl_2_·4H_2_O was dissolved in 10 mL of ethanolamine under vigorous stirring at room temperature, followed by adding 10 mL of deionized water. Then, the mixture was stirred at room temperature for 24 h. The obtained precipitates were washed with deionized water and ethanol. The final products were dried at 40 ^o^C under vacuum overnight.

### Synthesis of MoS_2_ nanosheets

MoS_2_ nanosheets were prepared by a reported method^33^. Briefly, (NH_4_)_6_Mo_7_O_24_·4H_2_O (1 mmol) and thiourea (30 mmol) were dissolved in deionized water (35 mL) under vigorous stirring to form a homogeneous solution. Then, the solution was transferred into a 45-mL Teflon-lined stainless steel autoclave and maintained at 220 ^o^C. After 18 h, the reaction system was allowed to cool down to room temperature. The final products were washed with deionized water and ethanol for several times. The final products were dried at 40 ^o^C under vacuum overnight.

### Synthesis of Co_0.8_Fe_0.2_Se_2_ nanosheets

The synthesis of Co_0.8_Fe_0.2_Se_2_ nannosheets was based on the selenization of cobalt-iron hydroxides^34^. First, CoCl_2_·6H_2_O (1.6 mmol), FeCl_3_ (0.4 mmol), NaCl (10 mmol), and hexamethylenetetramine (12 mmol) were dissolved in a mixture of deionized water (200 mL) and ethanol (20 mL). The solution was magnetically stirred at 90 ^o^C for 2 h, and cooled down to room temperature. The as-obtained cobalt-iron hydroxides were washed three times with deionized water and ethanol. Next, the products were dispersed in a mixture of Se (3.75 mmol), NaOH (7.5 mmol), hydrazine (0.14 mL), and DMF (25 mL) for selenization. The mixed solution was finally transferred into a 50-mL autoclave. After keeping at 180 ^o^C for 1 h, Co_0.8_Fe_0.2_Se_2_ nannosheets were obtained. The molar ratio of Co:Fe was determined by inductively coupled plasma atomic emission spectroscopy (ICP-AES) with a value of 0.78:0.22. The growth of Co_0.8_Fe_0.2_Se_2_ nanosheets on Ni foam followed a two-step procedure. In the first stage, cobalt-iron hydroxides were in-situ grown on Ni foam. Typically, CoCl_2_·6H_2_O (1.6 mmol), FeCl_3_ (0.4 mmol), NaCl (10 mmol), and hexamethylenetetramine (12 mmol) were dissolved in a mixture of deionized water (200 mL) and ethanol (20 mL). Then, acid-treated Ni foam (1 cm × 2 cm) was added into the mixture. Next, cobalt-iron hydroxides were in-situ grown on Ni foam under magnetic stirring at 90 ^o^C for 2 h. In the second step, the as-obtained product was further selenized. Typically, the cobalt-iron hydroxides@Ni foam was transferred into a 50-mL autoclave containing a mixture of Se (3.75 mmol), NaOH (7.5 mmol), hydrazine (0.14 mL), and DMF (25 mL) for selenization. The autoclave was kept at 180 ^o^C for 1 h and then cooled down to room temperature. The final product was washed with ethanol for three times and dried for later use. The mass loadings of Co_0.8_Fe_0.2_Se_2_ nanosheets on Ni foam was determined to be 1 mg cm^−2^ by ICP-AES.

### Synthesis of SACs

Electrochemical depositions of single-atom M (M = Ir, Ru, Rh, Pd, Ag, Pt, Au, Fe, Co, Ni, Zn, V, Cr, Mn, and Cu) on supports were conducted using a linear sweep voltammetry method. The depositions were conducted in a standard three-electrode system. A carbon rod was used as the counter electrode. An Ag/AgCl electrode was used as the reference electrode. The SACs were deposited on the working electrode. The as-prepared Co(OH)_2_ nanosheets, MnO_2_ nanosheets, MoS_2_ nanosheets, and Co_0.8_Fe_0.2_Se_2_ nanosheets supports were first mixed with equally weighted active carbon. Then the mixture was loaded onto the glassy carbon electrode by Nafion with a yield loading of 0.28 mg cm^−2^ as the working electrodes. N-C was loaded onto the glassy carbon electrode by Nafion with a yield loading of 0.56 mg cm^−2^ as the working electrode. Co_0.8_Fe_0.2_Se_2_@Ni foam was directly used as the working electrode. The electrolyte contains 100 μM metal precursors and 1 M KOH. IrCl_4_·xH_2_O, RuCl_3_, Na_3_RhCl_6_·12H_2_O, Na_2_PdCl_4_, AgNO_3_, H_2_PtCl_4_, HAuCl_4_·xH_2_O, FeCl_3_, CoCl_2_·6H_2_O, NiCl_2_, Zn(NO_3_)_2_·6H_2_O, VCl_3_, CrCl_3_, MnCl_2_·4H_2_O, and CuCl_2_ were used as the metal precursors for Ir, Ru, Rh, Pd, Ag, Pt, Au, Fe, Co, Ni, Zn, V, Cr, Mn, and Cu, respectively. The electrochemical depositions were carried out from 0.10 V to −0.40 V for cathodic deposition and from 1.10 V to 1.80 V for anodic deposition with a sweeping rate of 5 mV s^−1^. The processes were repeated for ten times and three times in cathodic and anodic deposition, respectively. After the depositions, the electrode was washed with deionized water and directly used for later electrochemical measurements. The metal mass loadings of these as-prepared SACs are shown in Table. S1. The electrochemical deposition of Ir single atoms was also carried out in acidic media using 0.5 M H_2_SO_4_ containing 100 μM IrCl_4_ precursors as the electrolyte, and N-C as the support.

### XAFS measurements

XAFS spectra at Ir *L*_3_-edge were obtained at the BL14W1 beam line of Shanghai Synchrotron Radiation Facility operated at 3.5 GeV under “top-up” mode with a constant current of 220 mA. The XAFS data of C-Ir_1_/Co(OH)_2_ and A-Ir_1_/Co(OH)_2_ were recorded under fluorescence mode with a 32-element Ge solid-state detector. The energy was calibrated according to the absorption edge of pure Ir powder. Athena and Artemis codes were used to extract the data and fit the profiles. For the XANES part, the experimental absorption coefficients as a function of energies *μ*(*E*) were processed by background subtraction and normalization procedures. We refer to this process as “normalized absorption”. For the EXAFS part, the Fourier-transformed data in *R* space were analyzed by applying the first shell approximation or metallic Ir model for the Ir-O or Ir-Ir shell, respectively. The passive electron factors, *S*_*0*_^2^, were determined by fitting the experimental Ir powder data and fixing the Ir-Ir coordination number (*CN*) to be 12. The determined factors were fixed for further analysis of the measured samples. Other parameters such as *CN*, bond distance (*R*), and Debye-Waller (*D. W.*) factor around the absorbed atoms were allowed to vary during the fitting process. XAFS spectra of other SACs were obtained at the 1W1B beam line of Beijing Synchrotron Radiation Facility.

### In-situ DRIFT

In-situ diffuse reflectance infrared Fourier transform (DRIFT) experiments of CO adsorption were conducted on a Fourier transform infrared spectrometer (TENSOR II Sample Compartment RT-DLaTGS). The DRIFT spectra of C-Rh_1_/Co(OH)_2_ were collected with a resolution of 8 cm^−1^ and 64 scans in Kubelka-Munk units at 100 °C. After flowing with He (30 sccm), the background spectrum was acquired when the background was stable. Then, CO (10 sccm) was allowed to flow into the cell until the gaseous CO peak intensity stopped growing (~20 min), suggesting saturated adsorption on the catalyst surfaces. In the end, only He (30 sccm) were used to purge out the gaseous CO from the sample cell so that the chemically adsorbed CO species on the samples could be detected. The DRIFT spectra of C-Pd_1_/Co(OH)_2_, C-Pt_1_/Co(OH)_2_, and C-Au_1_/Co(OH)_2_ were collected using the same procedures except for the temperature was set at 30 °C.

### Electrochemical measurements

An electrochemical workstation (CHI 660E, Shanghai CH Instruments) was used to measure the electrocatalytic properties of the samples. HER and OER electrocatalysis were measured in a standard three-electrode system at room temperature. The glassy carbon electrode loaded with the as-obtained catalysts was used as the working electrode. A carbon rod was used as the counter electrode. An Ag/AgCl electrode was used as the reference electrode. The polarization curves were obtained using a linear sweep voltammetry method. All potentials unless specially mentioned in this work were measured against the Ag/AgCl electrode and converted to reversible hydrogen electrode (RHE) scale by *E* (vs. RHE) = *E* (vs. Ag/AgCl) + 0.194 V + 0.059 pH V, where 0.194 V was obtained by calibrating the Ag/AgCl electrode against the RHE in 0.5 M H_2_SO_4_. The calibration was carried out using a three-electrode system in a high-purity hydrogen-saturated 0.5 M H_2_SO_4_ electrolyte using a cyclic voltammetry method with a sweep rate of 1 mV s^−1^. The Ag/AgCl was used as the reference electrode. Pt wires were used as both the working and counter electrodes. The average of the two potentials at zero current was taken to be the thermodynamic potential for the hydrogen electrode reactions. The HER polarization curves were recorded from 0.10 to −0.40 V with a sweeping rate of 5 mV s^−1^ in hydrogen-saturated 1 M KOH electrolyte. The OER polarization curves were recorded from 1.10 to 1.80 V with a sweeping rate of 5 mV s^−1^ in oxygen-saturated 1 M KOH electrolyte. The potentials were referred to the value *E*_*iR-*corrected_ after *iR*-correction by *E*_*iR-*corrected_ = *E* (vs. RHE) – *iR*, where *i* is the current, and *R* is the uncompensated ohmic electrolyte resistance. In this case, *R* was measured to be 9 Ω by high-frequency alternating-current impedance. Electrochemical impedance spectroscopy (EIS) for HER was conducted with alternating-current voltage with 5 mV amplitude at the potential of −0.20 V vs. RHE within the frequency range from 100 KHz to 100 mHz. Electrochemical impedance spectroscopy (EIS) for OER was conducted with alternating-current voltage with 5 mV amplitude at the potential of 1.53 V vs. RHE within the frequency range from 100 KHz to 100 mHz. The stability tests of Co_0.8_Fe_0.2_Se_2_@Ni foam towards HER and OER were conducted in a three-electrode system, where Hg/HgO electrode was used as the reference electrode. The potentials were converted to RHE by *E* (vs. RHE) = *E* (vs. Hg/HgO) + 0.098 V + 0.059 pH V, where 0.098 V was obtained by calibrating the Hg/HgO electrode against the RHE in 1 M KOH.

Overall water splitting electrocatalysis was conducted in a two-electrode cell. C-Ir_1_/Co_0.8_Fe_0.2_Se_2_@Ni foam and A-Ir_1_/Co_0.8_Fe_0.2_Se_2_@Ni foam with an area of 1 cm × 0.5 cm were used as cathode and anode, respectively. For comparison, commercial Pt/C-IrO_2_@Ni foam and Co_0.8_Fe_0.2_Se_2_@Ni foam were also coupled for catalyzing overall water splitting. IrO_2_ was loaded on active carbon with a mass loading of 20% to form IrO_2_/C. Both Pt/C and IrO_2_/C were loaded onto Ni foam with a mass loading of 1 mg cm^−2^. Pt/C on Ni foam was used as the cathode and IrO_2_/C on Ni foam was used as the anode. The polarization curves for overall water splitting were obtained using a linear sweep voltammetry method. The applied potential was set to range from 1.0 V to 2.0 V with a sweeping rate of 5 mV s^−1^. The potentials were corrected by *E*_*iR-*corrected_ = *E* – *iR*, where *R* was measured to be 1.8 Ω. Durability tests were conducted in galvanostatic mode at 10, 100, and 500 mA cm^−2^ at room temperature in 1 M KOH solution. As for the measurement of Faradaic efficiency (FE), the gaseous products were analyzed by gas chromatography. The production of H_2_ and O_2_ were measured separately in an H-type cell containing 1 M KOH electrolyte at a current density of 10 mA cm^−2^ for 140 min. In the measurement of Faradic efficiency for HER, the working electrode (C-Ir_1_/Co_0.8_Fe_0.2_Se_2_@Ni foam) and reference electrode (Ag/AgCl) were placed in the cathodic chamber. The counter electrode (carbon rod) was placed in the anodic chamber. During electrocatalysis, a flow of Ar at 20 sccm was continuously sparged into the cathodic chamber as carrier gas. In the measurement of Faradic efficiency for OER, working electrode (A-Ir_1_/Co_0.8_Fe_0.2_Se_2_@Ni foam) and reference electrode (Ag/AgCl) were placed in the anodic chamber. The counter electrode (carbon rod) was placed in the cathodic chamber. During electrocatalysis, a flow of Ar at 20 sccm was continuously sparged into the anodic chamber as carrier gas.

### Instrumentations

TEM images were taken on Hitachi H-7650 transmission electron microscope operating at an acceleration voltage of 100 kV. HAADF-STEM images and energy-dispersive X-ray (EDX) elemental mapping were carried out on JEOL ARM-200F field-emission transmission electron microscope operating at an accelerating voltage of 200 kV using Cu-based TEM grids. X-ray diffraction (XRD) patterns were recorded using a Philips X’Pert Pro Super diffractometer with Cu-Kα radiation (*λ* = 1.54178 Å). XPS measurements were performed on a VG ESCALAB MK II X-ray photoelectron spectrometer with Mg Kα = 1253.6 eV as the exciting source. Ultraviolet–Visible (UV) adsorption spectrum was conducted on UV–Vis spectrophotometer (Agilent Technologies, Cary 60). ICP-AES (Atomscan Advantage, Thermo Jarrell Ash, USA) analyses were used to determine the mass loading of metal species. scanning electron microscope (SEM) image and energy-dispersive X-ray spectra were taken on a scanning electron microscope (SEM, JSM-6700F) operated at 65 kV. The gas products of water splitting were monitored by an online GC (SHIMADZU, GC-2014) equipped with a thermal conductivity detector and Molsieve 5 A colum.

## Supplementary information

Supplementary Information

## Data Availability

The data that support the findings of this study are available from the corresponding author on reasonable request.
