# Supplementary material for: Electrochemical deposition as a universal route for fabricating single-atom catalysts
Source: Nat Commun. 2020 Mar 5;11:1215. doi: 10.1038/s41467-020-14917-6 (PMC7058015; doi:10.1038/s41467-020-14917-6)
Supplement: Supplementary file 1 — Supplementary Information [file 41467_2020_14917_MOESM1_ESM.pdf]

Supplementary Information for

Electrochemical deposition as a universal route for fabricating single-atom catalysts

Zhang et al.

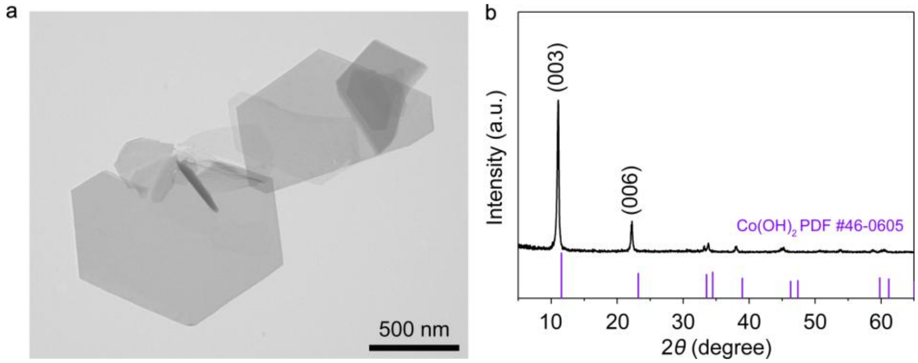

Supplementary Figure 1. Morphological and structural characterizations of $Co(OH)_2$ nanosheets. a, **b**, TEM image (**a**) and XRD pattern (**b**) of $Co(OH)_2$ nanosheets.

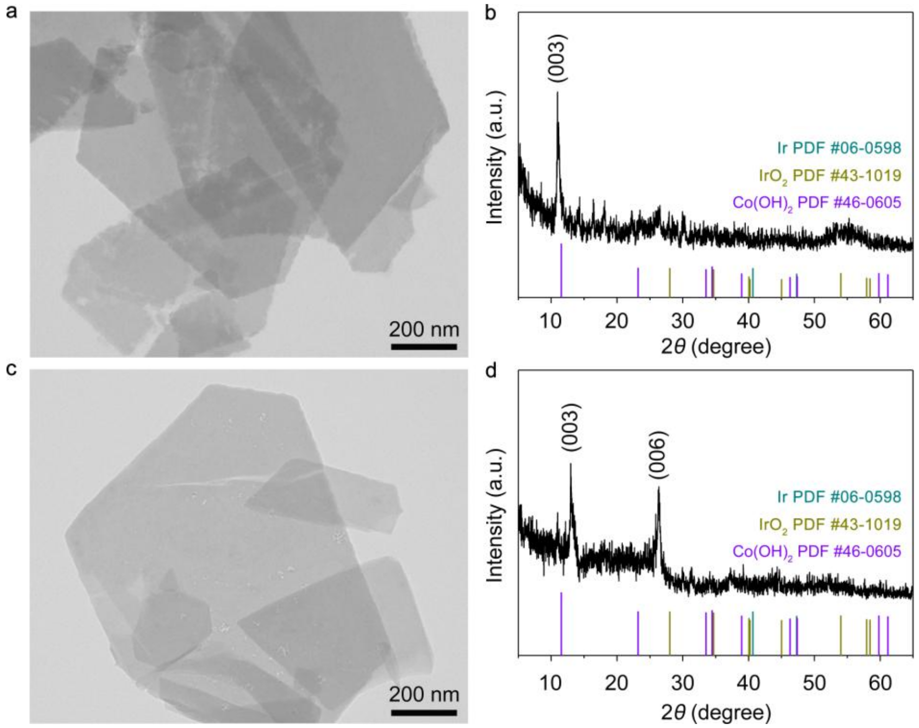

Supplementary Figure 2. Morphological and structural characterizations of $Ir_1/Co(OH)_2$. a, **b**, TEM image (**a**) and XRD pattern (**b**) of C-$Ir_1/Co(OH)_2$. **c**, **d**, TEM image (**c**) and XRD pattern (**d**) of A-$Ir_1/Co(OH)_2$.

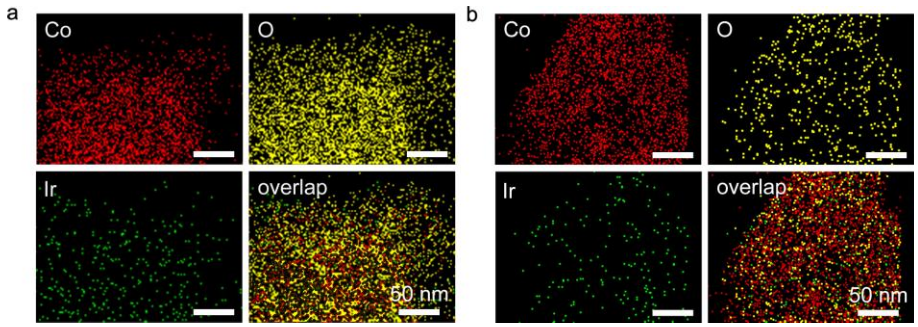

Supplementary Figure 3. Elemental distribution in $Ir_1/Co(OH)_2$. a, **b**, Energy-dispersive X-ray elemental mapping images of C-$Ir_1/Co(OH)_2$ (**a**) and A-$Ir_1/Co(OH)_2$ (**b**).

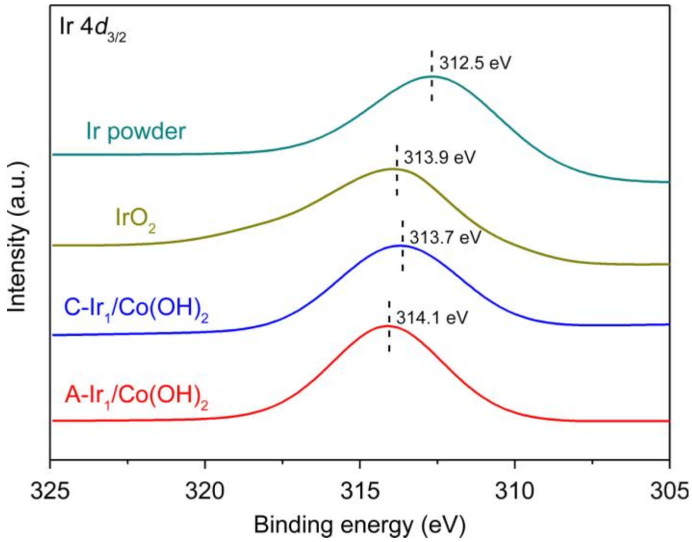

Supplementary Figure 4. Ir 4*d* XPS spectra of C-$Ir_1/Co(OH)_2$ and A-$Ir_1/Co(OH)_2$. Ir powder and IrO_2 were used as references.

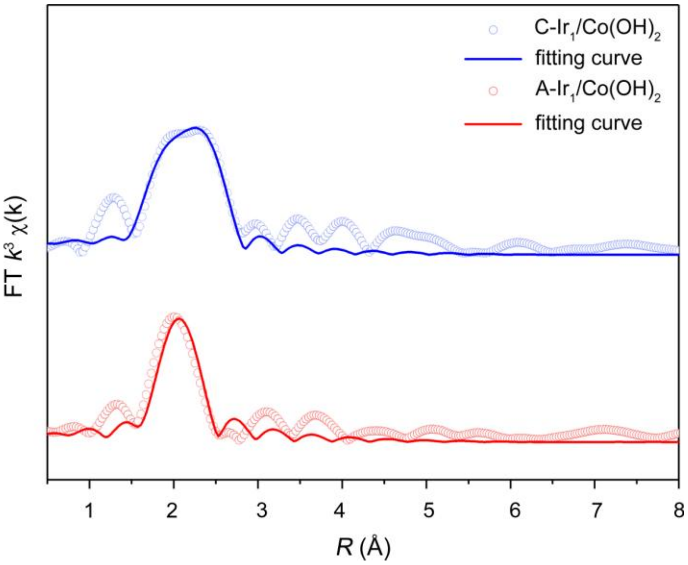

Supplementary Figure 5. EXAFS data and fitting results of C-$Ir_1/Co(OH)_2$ and A-$Ir_1/Co(OH)_2$.

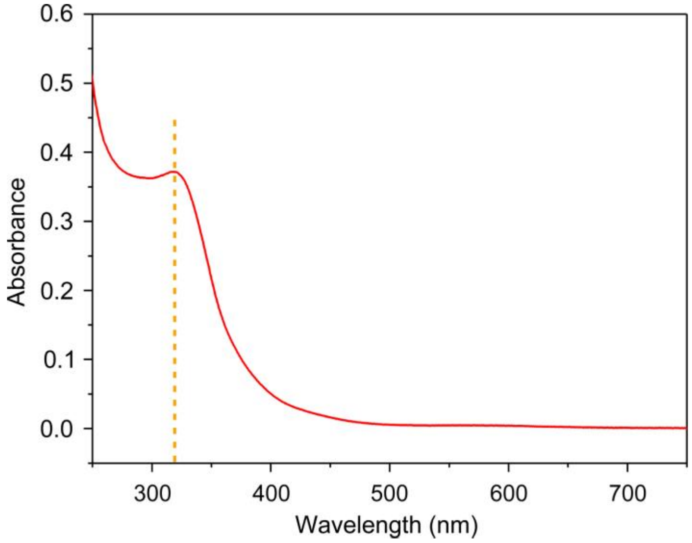

Supplementary Figure 6. UV-vis spectrum of the electrolyte containing 1 M KOH and 100 μM $IrCl_4$. A notable peak is shown at 318 nm, which is ascribed to the adsorption of $Ir(OH)_6^{2-}$.

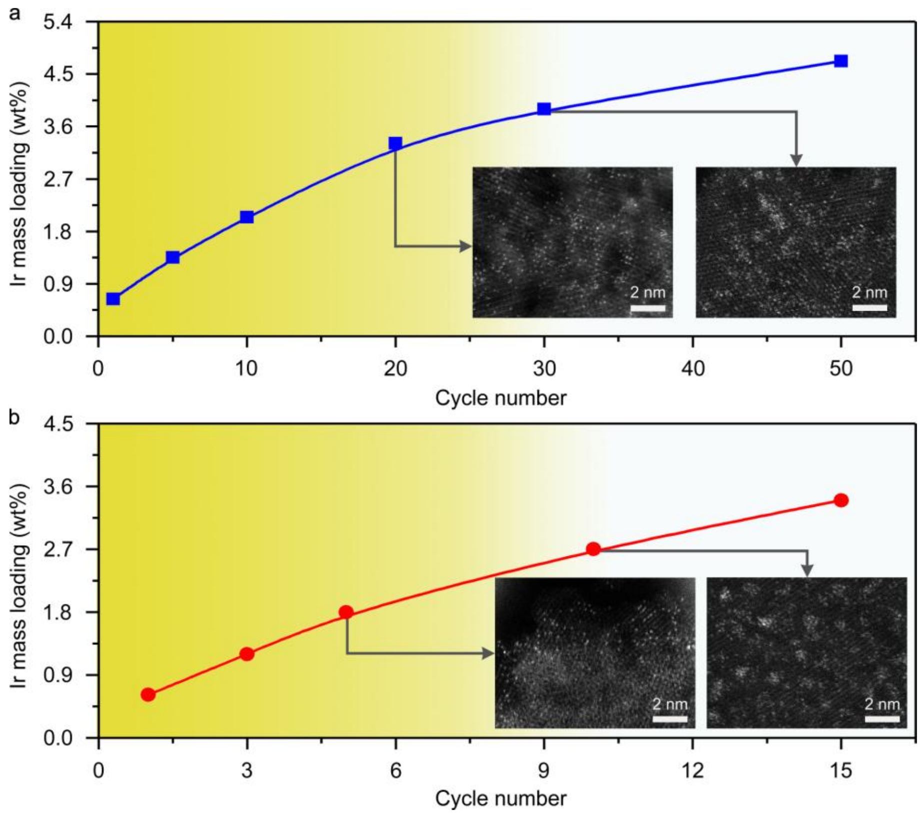

Supplementary Figure 7. The influence of scanning cycles on the formation of SACs. a, **b**, Ir mass loading as a function of scanning cycles in cathodic deposition (**a**) and anodic deposition (**b**). The depositions were conducted in 1 M KOH electrolyte containing 100 μM Ir precursors. The inset images correspond to the HAADF-STEM images of the samples obtained after a certain number of scanning cycles.

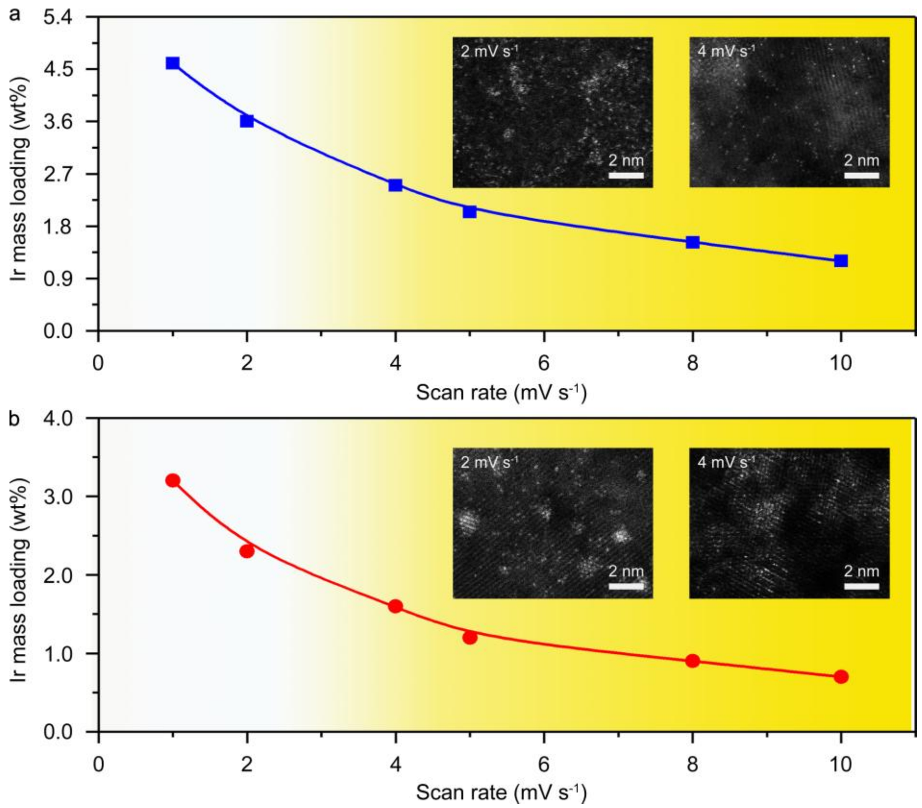

Supplementary Figure 8. The influence of scanning rate on the formation of SACs. a, **b**, Ir mass loading as a function of scanning rate in cathodic (**a**) and anodic deposition (**b**). The depositions were conducted in the 1 M KOH electrolyte containing 100 μM Ir precursors. The scanning cycle number was kept at ten for cathodic deposition and three for anodic deposition. The color gradient from yellow to white indicates the transition from single atoms to clusters with decreasing scanning rate. The inset images correspond to the HAADF-STEM images of the samples obtained at a certain scanning rate.

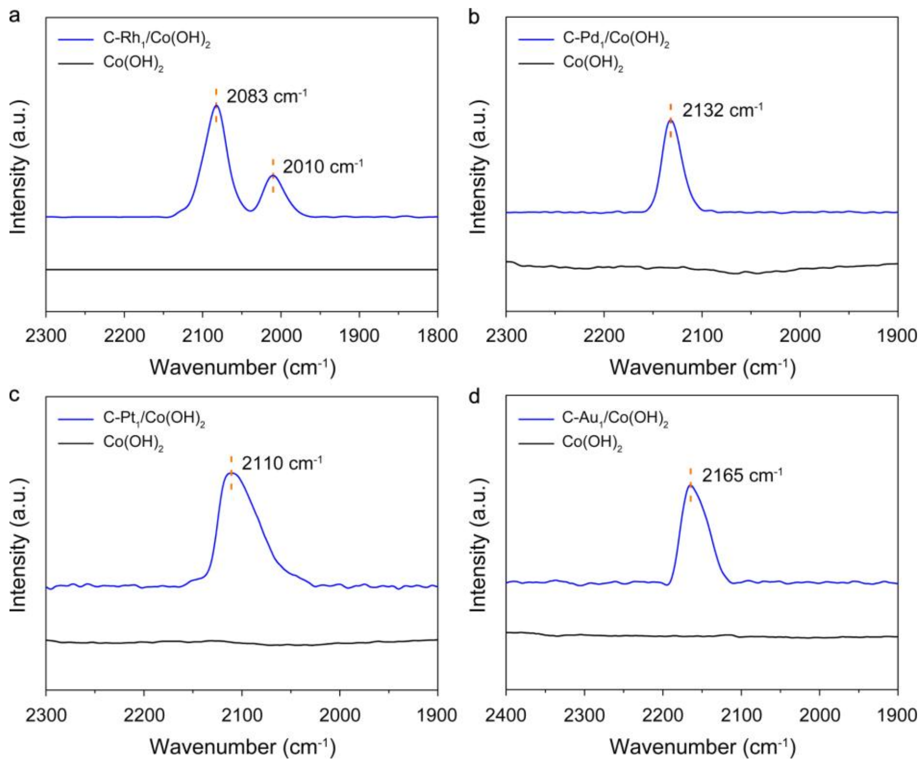

Supplementary Figure 9. *In-situ* DRIFT spectra of CO adsorption for (**a**) C-$Rh_1/Co(OH)_2$, (**b**) C-$Pd_1/Co(OH)_2$, (**c**) C-$Pt_1/Co(OH)_2$, and (**d**) C-$Au_1/Co(OH)_2$. For C-$Rh_1/Co(OH)_2$, two prominent peaks showed at 2083 and 2010 cm^{-1}, ascribing to the symmetric and asymmetric stretching vibrations of CO from the isolated mononuclear $Rh_1(CO)_2$ species, respectively[1]. For C-$Pd_1/Co(OH)_2$, the peak at 2132 cm^{-1} is attributed to atop CO adsorption on singly dispersed Pd species[2]. For C-$Pt_1/Co(OH)_2$, the peak at 2110 cm^{-1} is assigned to CO linearly adsorbed on atomic Pt species[3]. For C-$Au_1/Co(OH)_2$, only a dominant peak showed at 2165 cm^{-1}, ascribing to mononuclear Au species adsorbed on CO[4].

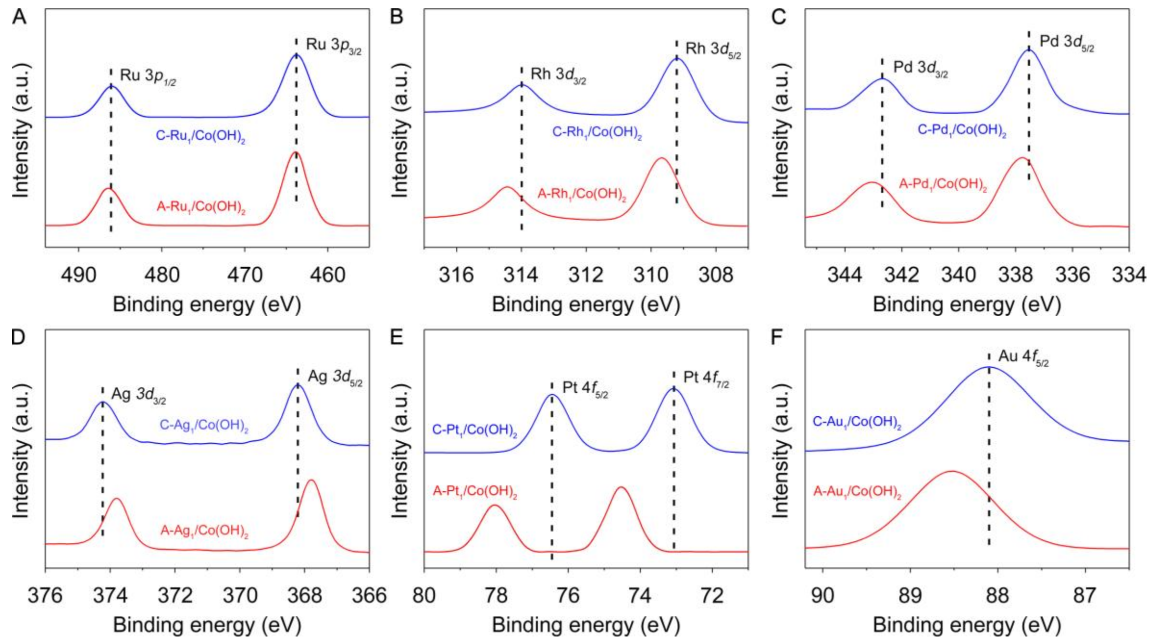

Supplementary Figure 10. XPS spectra of different SACs. a, Ru 3*p* XPS spectra of C-$Ru_1/Co(OH)_2$ and A-$Ru_1/Co(OH)_2$. **b**, Rh 3*d* XPS spectra of C-$Rh_1/Co(OH)_2$ and A-$Rh_1/Co(OH)_2$. **c**, Pd 3*d* XPS spectra of C-$Pd_1/Co(OH)_2$ and A-$Pd_1/Co(OH)_2$. **d**, Ag 3*d* XPS spectra of C-$Ag_1/Co(OH)_2$ and A-$Ag_1/Co(OH)_2$. **e**, Pt *4f* XPS spectra of C-$Pt_1/Co(OH)_2$ and A-$Pt_1/Co(OH)_2$. **f**, Au *4f* XPS spectra of C-$Au_1/Co(OH)_2$ and A-$Au_1/Co(OH)_2$.

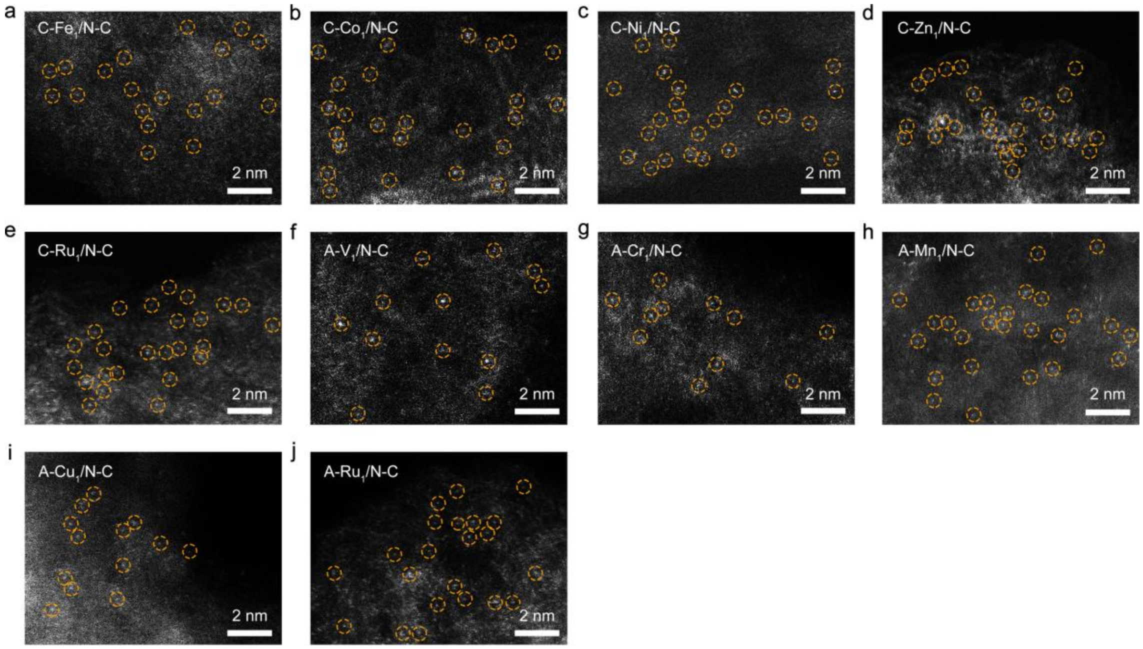

Supplementary Figure 11. HAADF-STEM images of various SACs. a-e, HAADF-STEM images of cathodically deposited C-Fe_1/N-C (**a**), C-Co_1/N-C (**b**), C-Ni_1/N-C (**c**), C-Zn_1/N-C (**d**), and C-Ru_1/N-C (**e**). **f**-**j**, HAADF-STEM images of anodically deposited A-V_1/N-C (**f**), A-Cr_1/N-C (**g**), A-Mn_1/N-C (**h**), A-Cu_1/N-C (**i**), and A-Ru_1/N-C (**j**). The depositions were all conducted in a 1 M KOH electrolyte containing 100 μM metal precursors. For cathodic deposition, the deposition process was conducted in a potential ranging from 0.10 to -0.40 V for ten scanning cycles. For anodic deposition, the deposition process was conducted in a potential ranging from 1.10 to 1.80 V for three scanning cycles.

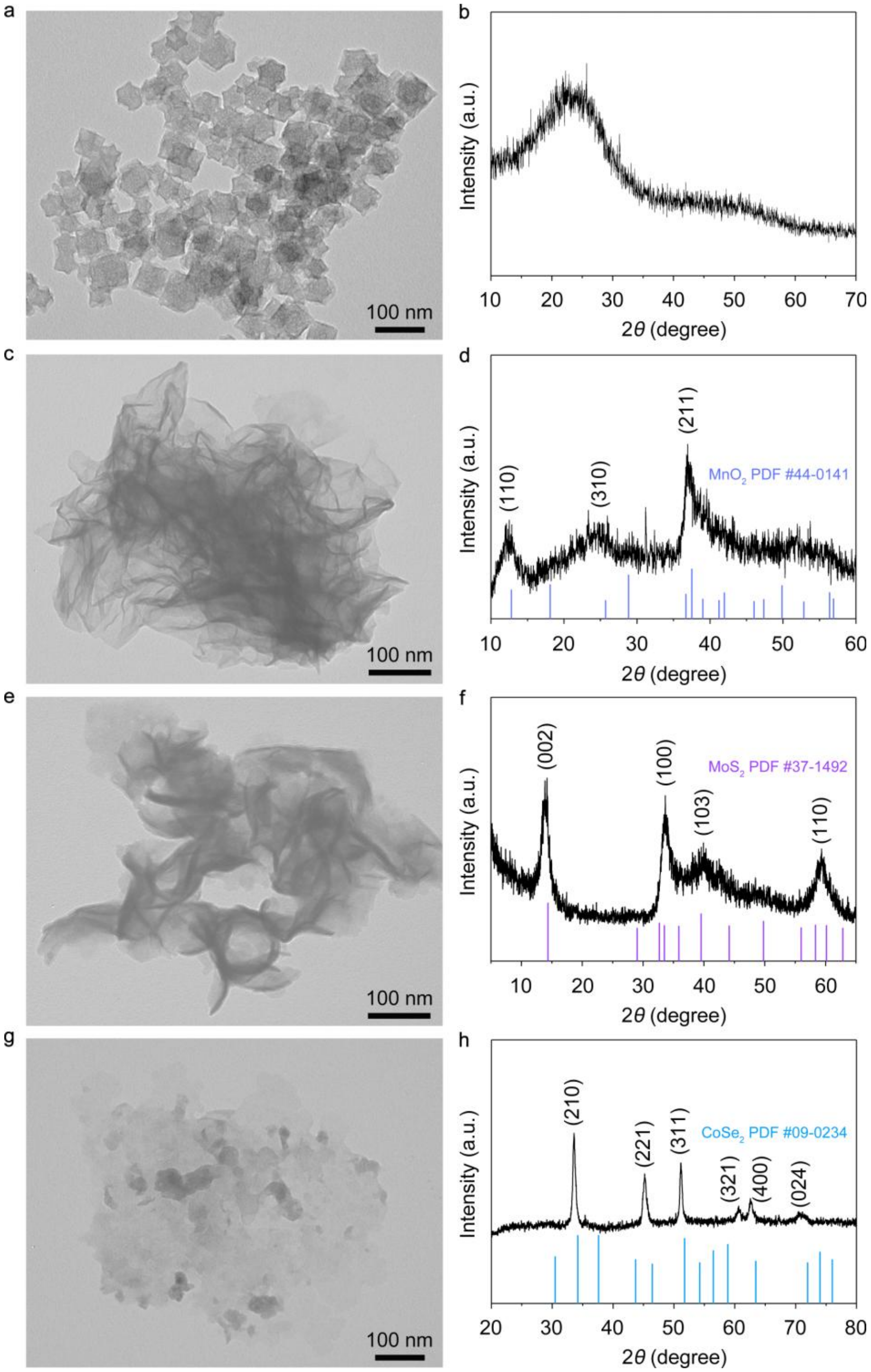

Supplementary Figure 12. Morphological and structural characterizations of different supports. a, **b**, TEM image (**a**) and XRD pattern of N-C (**b**). **c**, **d**, TEM image (**c**) and XRD pattern (**d**) of MnO_2 nanosheets. **e**, **f**, TEM image (**e**) and XRD pattern (**f**) of MoS_2 nanosheets. **g**, **h**, TEM image (**g**) and XRD pattern (**h**) of $Co_{0.8}Fe_{0.2}Se_2$ nanosheets.

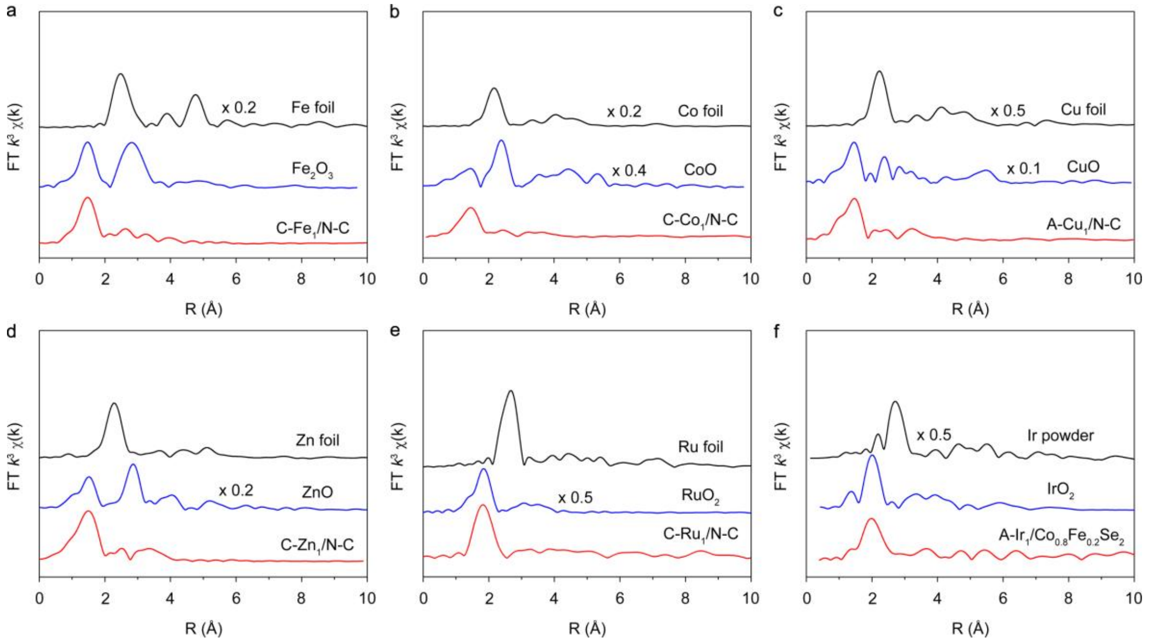

Supplementary Figure 13. EXAFS spectra of different SACs. a, EXAFS spectra of C-Fe_1/N-C at the Fe *K*-edge. Fe foil and Fe_2O_3 were used as references. **b**, EXAFS spectra of C-Co_1/N-C at the Co *K*-edge. Co foil and CoO were used as references. **c**, EXAFS spectra of A-Cu_1/N-C at the Cu *K*-edge. Cu foil and CuO were used as references. **d**, EXAFS spectra of C-Zn_1/N-C at the Zn *K*-edge. Zn foil and ZnO were used as references. **e**, EXAFS spectra of C-Ru_1/N-C at the Ru *K*-edge. Ru foil and RuO_2 were used as references. **f**, EXAFS spectra of A-Ir_1/$Co_{0.8}Fe_{0.2}Se_2$ at the Ir L_3-edge. Ir powder and IrO_2 were used as references.

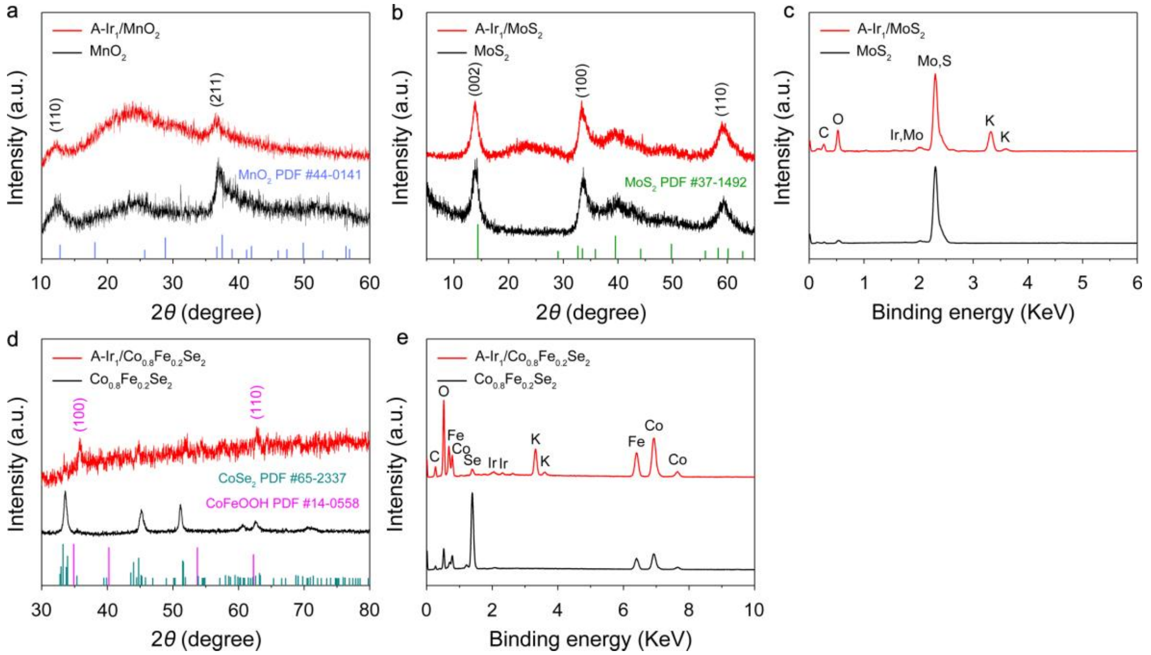

Supplementary Figure 14. Structural and compositional characterizations of different supports before and after deposition single atoms. **a**, XRD patterns of MnO_2 and A-Ir_1/MnO_2. **b**, **c**, XRD patterns (**b**) and EDS spectra (**c**) of MoS_2 and A-Ir_1/MoS_2. **d**, **e**, XRD patterns (**d**) and EDS spectra (**e**) of $Co_{0.8}Fe_{0.2}Se_2$ and A-$Ir_1/Co_{0.8}Fe_{0.2}Se_2$. The arise of K peaks in the EDS spectra of A-Ir_1/MoS_2 and A-$Ir_1/Co_{0.8}Fe_{0.2}Se_2$ was due to the adsorption of K^+ after the electrodeposition in 1 M KOH electrolyte.

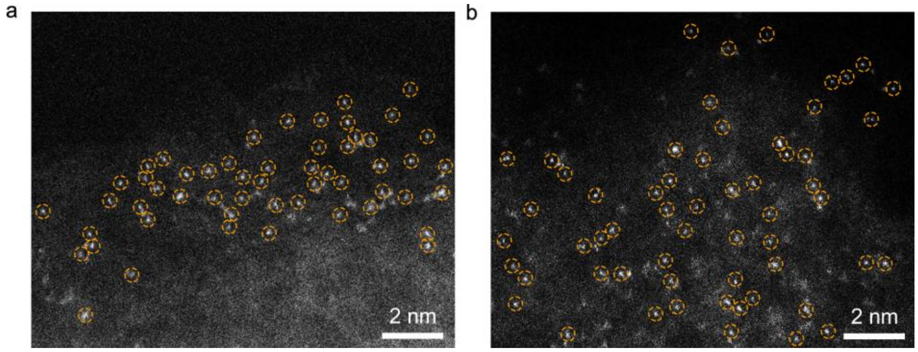

Supplementary Figure 15. Structural and morphological characterizations of SACs deposited in acid media. a, **b**, HAADF images of cathodically (**a**) and anodically (**b**) deposited Ir single atoms on N-C in 0.5 M H_2SO_4 electrolyte containing 100 μM $IrCl_4$. For cathodic deposition, the deposition process was conducted in a potential range from 0.1 to -0.4 V (vs RHE) for three scanning cycles. For anodic deposition, the deposition process was conducted in a potential range from 1.1 to 1.8 V (vs RHE) for ten scanning cycles. The singly-dispersed Ir atoms are marked by yellow circles.

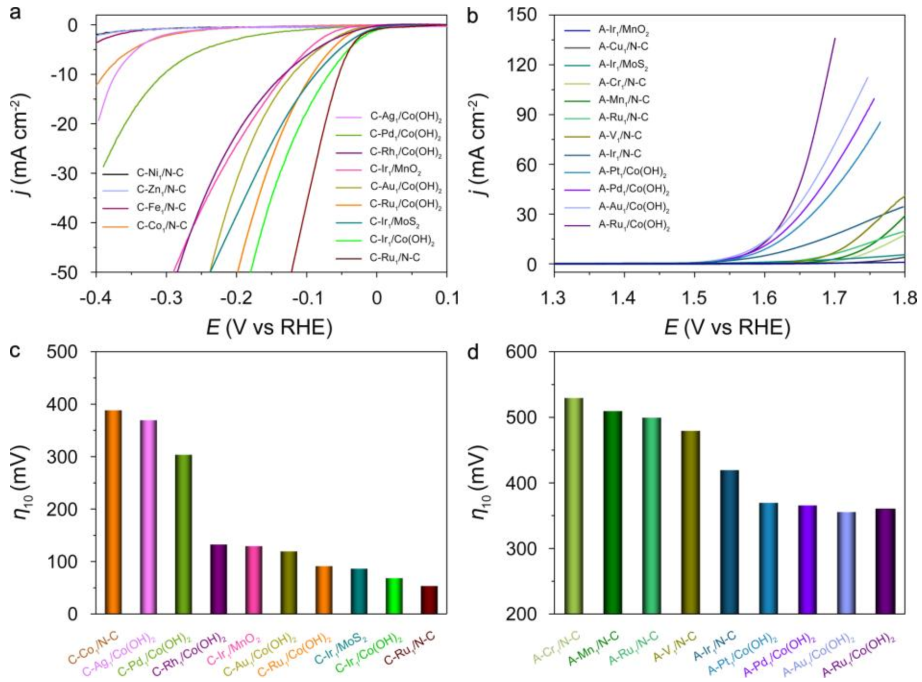

Supplementary Figure 16. Electrocatalytic performances of SACs for water splitting. a, **b**, Polarization curves of cathodically deposited SACs for HER (**a**) and anodically deposited SACs for OER (**b**). **c**, **d**, The overpotentials at 10 mA cm^{-2} for HER (**c**) and OER (**d**). Due to the poor performance of C-Ni_1/N-C, C-Zn_1/N-C, and C-Fe_1/N-C for HER and A-Ir_1/MnO_2, A-Cu_1/N-C, and A-Ir_1/MoS_2 for OER, the current densities of these catalysts did not reach 10 mA cm^{-2} in the potential range of our tests. Therefore, their overpotentials at 10 mA cm^{-2} (η_{10}) were not given.

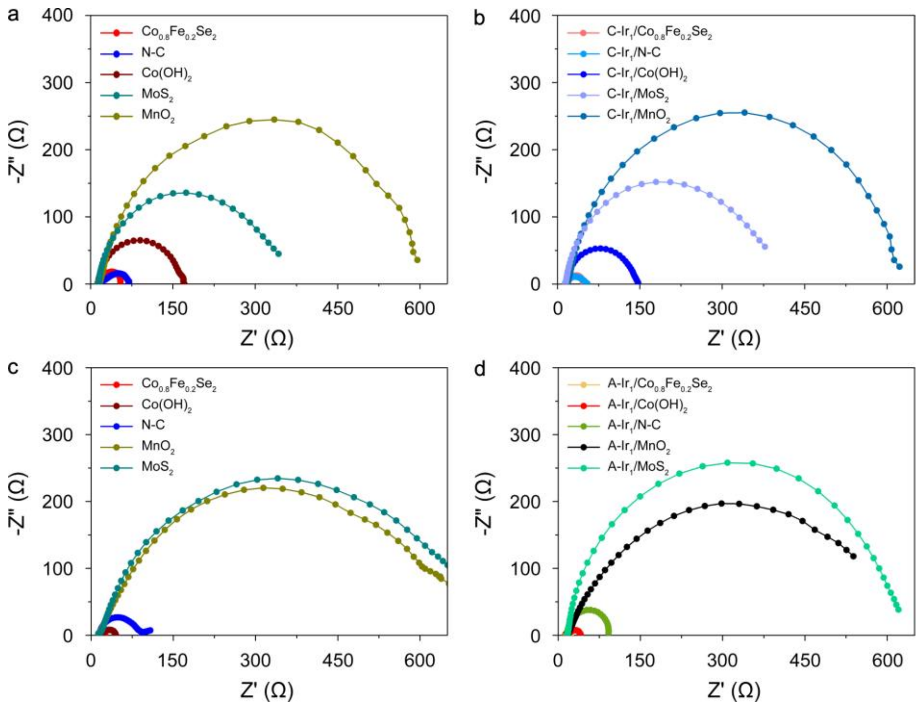

Supplementary Figure 17. EIS spectra for HER and OER. a, **b**, EIS spectra of different substrates (**a**) and cathodically deposited Ir single atoms on different substrates (**b**) for HER at the potential of -0.20 V. **c**, **d**, EIS spectra of different substrates (**c**) and anodically deposited Ir single atoms on different substrates (**d**) for OER at the potential of 1.53 V.

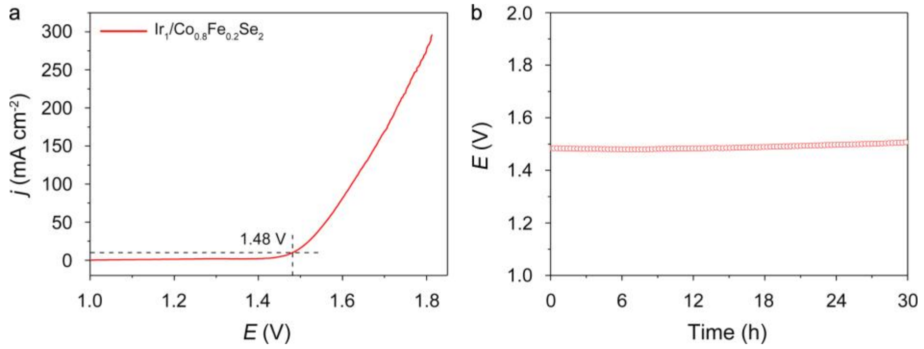

Supplementary Figure 18. Electrocatalytic performances of $Ir_1/Co_{0.8}Fe_{0.2}Se_2$ for overall water splitting. **a**, Polarization curve. **b**, Chronopotentiometric curve at 10 mA cm^{-2}.

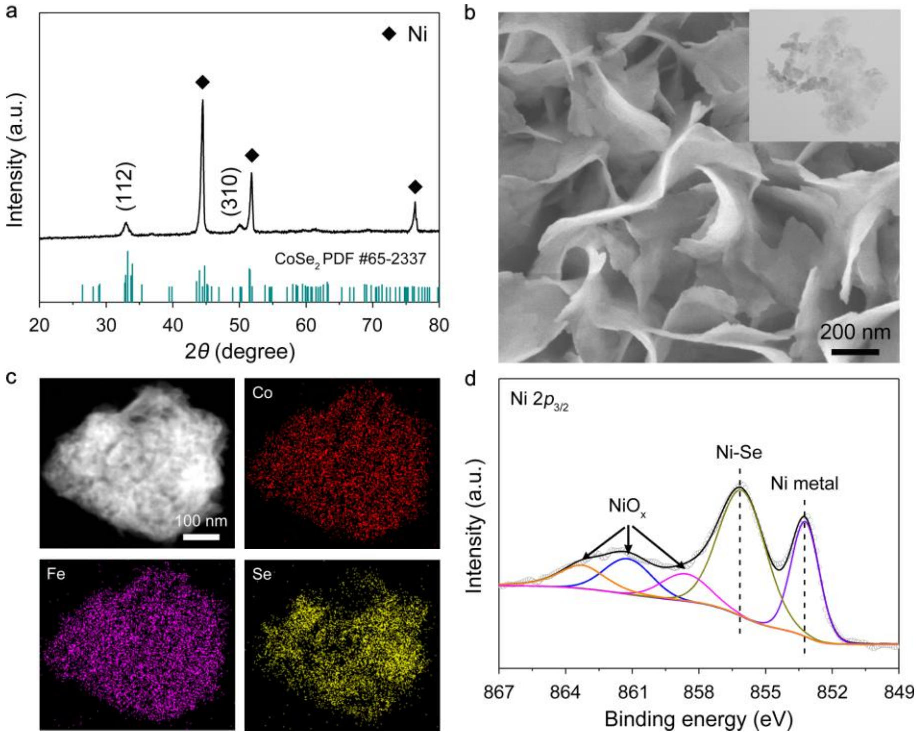

Supplementary Figure 19. Structural and morphological characterizations of $Co_{0.8}Fe_{0.2}Se_2$@Ni foam. **a**, XRD pattern of $Co_{0.8}Fe_{0.2}Se_2$@Ni foam. **b**, SEM image of $Co_{0.8}Fe_{0.2}Se_2$@Ni foam. The inset shows the TEM image of $Co_{0.8}Fe_{0.2}Se_2$. **c**, EDX elemental mapping images of $Co_{0.8}Fe_{0.2}Se_2$. To perform the TEM imaging and EDX elemental mapping characterizations, the $Co_{0.8}Fe_{0.2}Se_2$ nanosheets were exfoliated from $Co_{0.8}Fe_{0.2}Se_2$@Ni foam by ultrasonic treatment. **d**, Ni 2*p* XPS spectra and fitting curves of $Co_{0.8}Fe_{0.2}Se_2$@Ni foam. Three characteristic peaks at 863.3, 861.2, and 858.6 eV correspond to nickel oxides. The peak at 853.2 eV showed the existence of metallic Ni. The peak between nickel oxides and metallic Ni with a binding energy of 856.1 eV was assigned to nickel selenides. Combining the XRD pattern in **a**, the main phase of the Ni foam after the selenization process remained to be metallic Ni, while its surface was partially selenized. This may owe to that part of the Ni foam was not covered with cobalt-iron hydroxides before the selenization step and was then selenized, while the covered part remained to be Ni foam.

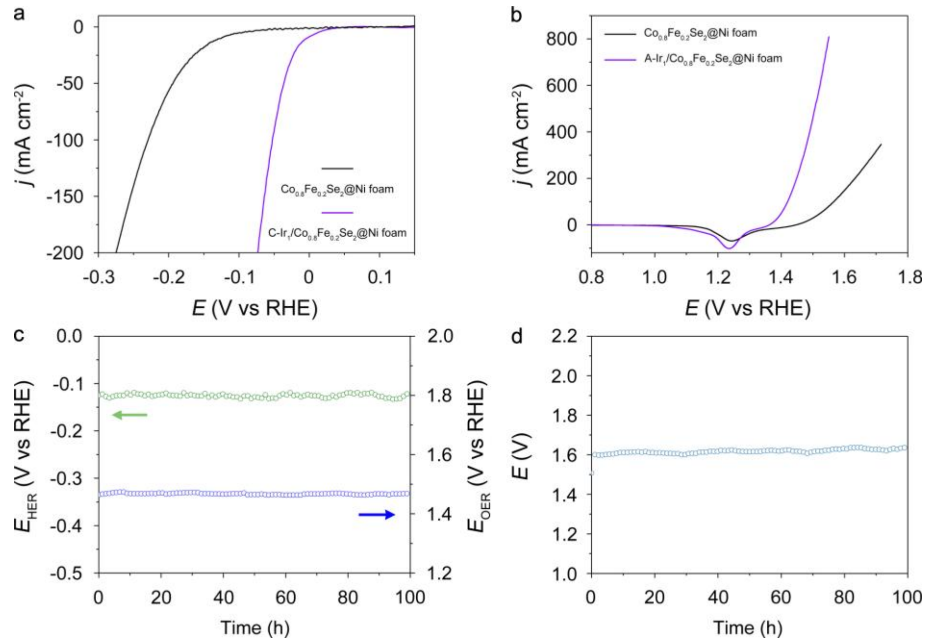

Supplementary Figure 20. Electrocatalytic performances of cathodic and anodic $Ir_1/Co_{0.8}Fe_{0.2}Se_2$@Ni foam for water splitting. a, Polarization curves of C-$Ir_1/Co_{0.8}Fe_{0.2}Se_2$@Ni foam and $Co_{0.8}Fe_{0.2}Se_2$@Ni foam for HER in a three-electrode system. **b**, Polarization curves of A-$Ir_1/Co_{0.8}Fe_{0.2}Se_2$@Ni foam and $Co_{0.8}Fe_{0.2}Se_2$@Ni foam for OER in a three-electrode system. **c**, Chronopotentiometric curves of $Co_{0.8}Fe_{0.2}Se_2$@Ni foam for HER and OER at 10 mA cm^{-2} over 100 h. **d**, Chronopotentiometric curves of $Co_{0.8}Fe_{0.2}Se_2$@Ni foam for overall water splitting at 10 mA cm^{-2} over 100 h.

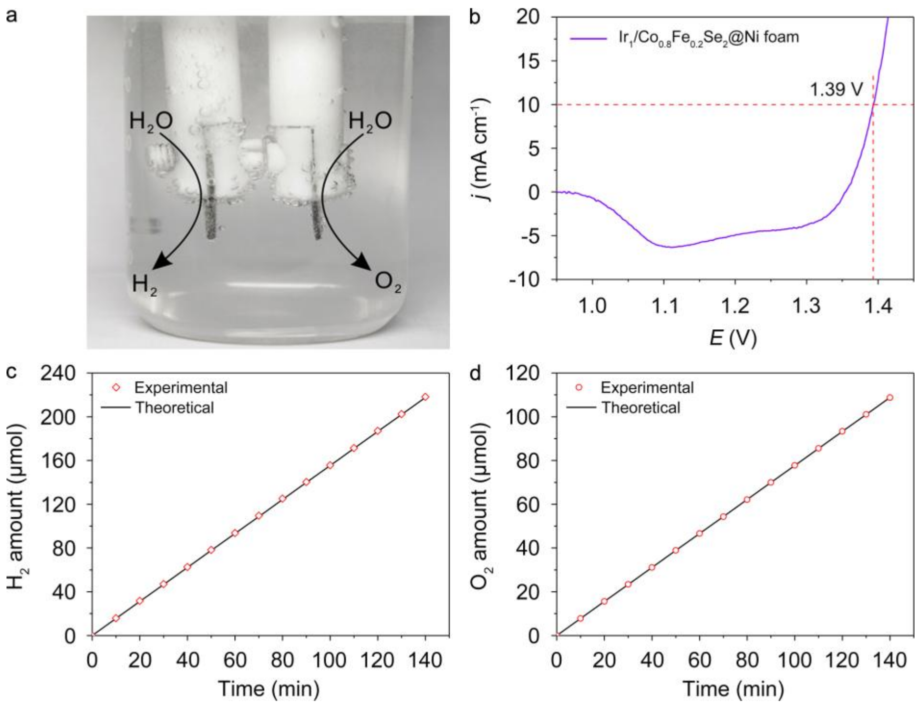

Supplementary Figure 21. Two-electrode cell and characterizations of overall water splitting. a, The two-electrode cell used for the stability test of $Ir_1/Co_{0.8}Fe_{0.2}Se_2$@Ni foam during overall water splitting. **b**, Magnified polarization curves of $Ir_1/Co_{0.8}Fe_{0.2}Se_2$@Ni foam for overall water splitting from 0 to 20 mA cm^{-2} in Figure 4e. **c**, **d**, Faradaic efficiency of $Ir_1/Co_{0.8}Fe_{0.2}Se_2$@Ni foam for HER and OER, respectively. The amount of H_2 generated over C-$Ir_1/Co_{0.8}Fe_{0.2}Se_2$@Ni foam (**c**) and O_2 generated over A-$Ir_1/Co_{0.8}Fe_{0.2}Se_2$@Ni foam (**d**) in comparison with the theoretical amount of gases at a current density of 10 mA cm^{-2} for 140 min.

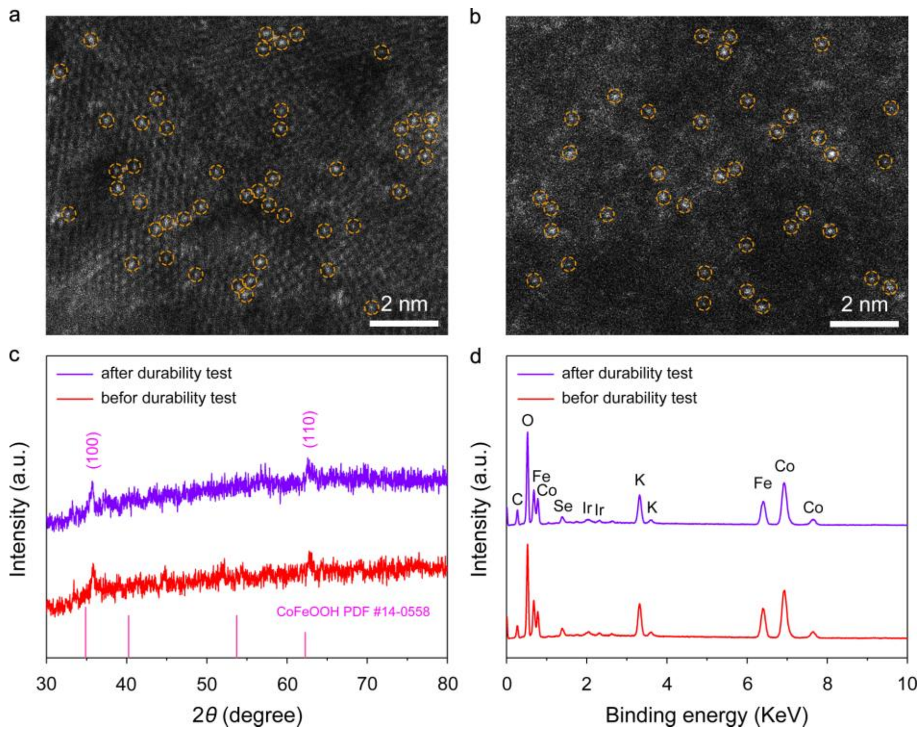

Supplementary Figure 22. Characterizations of the catalysts after durability tests toward overall water splitting at a current density of 10 mA cm^{-2} for 100 h. a, **b**, HAADF images of C-$Ir_1/Co_{0.8}Fe_{0.2}Se_2$@Ni foam (**a**) and A-$Ir_1/Co_{0.8}Fe_{0.2}Se_2$@Ni foam (**b**). The Ir single atoms in both catalysts showed no obvious change after the durability tests. (**c**) XRD patterns and (**d**) EDS spectra of A-$Ir_1/Co_{0.8}Fe_{0.2}Se_2$ before and after durability test for OER at 10 mA cm^{-2} over 100 h.

Supplementary Table 1. Metal mass loadings of electrochemically deposited SACs.

Samples	Metal Mass Loadings (wt%)	Samples	Metal Mass Loadings (wt%)
C-$Ir_1/Co(OH)_2$	2.0	A-$Ir_1/Co(OH)_2$	1.2
C-$Ru_1/Co(OH)_2$	1.2	A-$Ru_1/Co(OH)_2$	0.9
C-$Rh_1/Co(OH)_2$	1.3	A-$Rh_1/Co(OH)_2$	0.8
C-$Pd_1/Co(OH)_2$	1.0	A-$Pd_1/Co(OH)_2$	0.9
C-$Ag_1/Co(OH)_2$	0.9	A-$Ag_1/Co(OH)_2$	0.8
C-$Pt_1/Co(OH)_2$	1.5	A-$Pt_1/Co(OH)_2$	1.0
C-$Au_1/Co(OH)_2$	1.8	A-$Au_1/Co(OH)_2$	1.1
C-Fe_1/N-C	0.5	A-V_1/N-C	0.3
C-Co_1/N-C	0.7	A-Cr_1/N-C	0.3
C-Ni_1/N-C	0.6	A-Mn_1/N-C	0.5
C-Zn_1/N-C	0.6	A-Cu_1/N-C	0.4
C-Ru_1/ N-C	0.8	A-Ru_1/ N-C	0.7
C-Ir_1/MnO_2	1.1	A-Ir_1/MnO_2	0.8
C-Ir_1/MoS_2	1.3	A-Ir_1/MoS_2	0.9
C-$Ir_1/Co_{0.8}Fe_{0.2}Se_2$	2.3	A-$Ir_1/Co_{0.8}Fe_{0.2}Se_2$	1.8
C-Ir_1/N-C	1.6	A-Ir_1/N-C	1.4

All the SACs were obtained by cathodic or anodic deposition of metal precursors onto the supports. The depositions were conducted in a 1 M KOH electrolyte containing 100 μM metal precursors. For cathodic deposition, the deposition process was conducted in a potential ranging from 0.10 to -0.40 V for ten scanning cycles. For anodic deposition, the deposition process was conducted in a potential ranging from 1.10 to 1.80 V for three scanning cycles. The samples obtained from cathodic deposition were denoted with a capital "C", while those from anodic deposition were denoted with a capital "A".

Supplementary Table 2. EXAFS fitting results of C-$Ir_1/Co(OH)_2$ and A-$Ir_1/Co(OH)_2$. Ir powder and IrO_2 were used as references.

Samples	Ir-O		Ir-Cl		Ir-Ir		*D. W.*	ΔE_0 (eV)
	R (Å)	*CN*	*R* (Å)	*CN*	*R* (Å)	*CN*		
Ir powder	—	—	—	—	2.71	12	0.004	7.9±1.3
IrO_2	1.98±0.01	6	—	—	—	—	0.0024±0.0014	15.8±2.2
C-$Ir_1/Co(OH)_2$	1.97±0.02	3.3±0.9	2.32±0.02	3.1±0.4	—	—	0.003 (O)	13.3±3.2
A-$Ir_1/Co(OH)_2$	2.01±0.02	5.8±0.4	—	—	—	—	0.005 (Cl)	8.8±1.9

R, distance between absorber and backscatter atoms; *CN*, coordination number; *D. W.*, Debye-Waller factor; ΔE_0, inner potential correction that accounts for the difference in the inner potential between the sample and the references.

Supplementary Table 3. Comparison of HER performance for reported electrocatalysts in alkaline electrolytes.

Electrocatalysts	Electrolytes	Overpotential (mV) at j = 10 mA cm^{-2}	Ref.
**C-Ir$_1$/Co$_{0.8}$Fe$_{0.2}$Se$_2$**	**1 M KOH**	**8**	**This work**
Ru@Co-SAs/ N-doped carbon	1 M KOH	7	5
Ru@carbon quantum dots	1 M KOH	10	6
Co-substituted Ru	1 M KOH	13	7
Ru@nitrogenated holey two-dimensional carbon	1 M KOH	17	8
Ru/N-doped carbon	1 M KOH	21	9
Ru@ N-doped carbon	1 M KOH	26	10
RuCo@ N-doped carbon	1 M KOH	28	11
Ru-MoO$_2$	1 M KOH	29	12
Ru@N-doped carbon	1 M KOH	32	13
Pt/Co(OH)$_2$	1 M KOH	32	14
PtNi nanowires/C	1 M KOH	40	15
IrCo@N-doped carbon	1 M KOH	45	16
Hexagonal-PtNi	0.1 M KOH	65	17
Ru/C$_3$N$_4$/C	0.1 M KOH	79	18
Cu$_{2-x}$S@Ru	1 M KOH	82	19
MoC$_x$	1 M KOH	151	20
c-CoSe$_2$/carbon - cloth	1 M KOH	190	21

Supplementary Table 4. Comparison of overall water splitting performance for reported electrocatalysts in alkaline electrolytes.

**Electrocatalysts**	***j*** **(mA cm^{-2})**	**Overall voltage (V) at *j***	**Ref.**
**$Ir_1/Co_{0.8}Fe_{0.2}Se_2$@Ni foam**	**10**	**1.39**	**This work**
	**100**	**1.49**	
	**500**	**1.62**	
Co_3O_4@C	10	1.40	22
Co_3O_4 ultrathin nanosheet arrays	10	1.41	23
FeP/Ni_2P	10	1.42	24
	100	1.60	
FeCoNi-based LDH* nanowire arrays	10	1.429	25
Exfoliated FeCo LDH* @graphdiyne/Ni foam	10	1.43	26
Ni-Co complexes-MoS_2	10	1.44	27
NiFe LDH*-nanosheets @defective graphene10	10	1.44	28
MoO_x/Ni_3S_2/Ni foam	10	1.45	29
Cu@CoS_x/Co foam	10	1.50	30
	100	1.80	
$NiFeO_x$	10	1.51	31
Cu@NiFe LDH*	10	1.54	32
	100	1.69	
$FePO_4$/Ni foam	10	1.54	33
Three dimensional FeF_2-F_2O_3 nanoporous film	10	1.58	34
Se-(NiCo)S_x/$(OH)_x$	10	1.60	35

*LDH, layered double hydroxide.

Supplementary References

1. Kwon, Y., Kim, T. Y., Kwon, G., Yi, J. & Lee, H. Selective activation of methane on single-atom catalyst of rhodium dispersed on zirconia for direct conversion. *J. Am. Chem. Soc.* **139**, 17694-17699 (2017).
2. Liu, P. et al. Photochemical route for synthesizing atomically dispersed palladium catalysts. *Science* **352**, 797-800 (2016).
3. Nie, L. et al. Activation of surface lattice oxygen in single-atom Pt/CeO_2 for low-temperature CO oxidation. *Science* **358**, 1419-1423 (2017).
4. Lei, L. et al. Aerobic oxidation of alcohols over isolated single Au atoms supported on CeO_2 nanorods: Catalysis of interfacial [O–Ov–Ce–O–Au] sites. *ACS Appl. Nano Mater.* **2**, 5214-5223 (2019).
5. Yuan, S. et al. A universal synthesis strategy for single atom dispersed cobalt/metal clusters heterostructure boosting hydrogen evolution catalysis at all pH values. *Nano Energy* **59**, 472-480 (2019).
6. Li, W. et al. Carbon-quantum-dots-loaded ruthenium nanoparticles as an efficient electrocatalyst for hydrogen production in alkaline media. *Adv. Mater.* **30**, 1800676 (2018).
7. Mao, J. et al. Accelerating water dissociation kinetics by isolating cobalt atoms into ruthenium lattice. *Nat. Commun.* **9**, 4958 (2018).
8. Mahmood, J. et al. An efficient and pH-universal ruthenium-based catalyst for the hydrogen evolution reaction. *Nat. Nanotechnol.* **12**, 441-446 (2017).
9. Zhang, J. et al. Ruthenium/nitrogen-doped carbon as an electrocatalyst for efficient hydrogen evolution in alkaline solution. *J. Mater. Chem. A* **5**, 25314-25318 (2017).
10. Wang, Z.-L. et al. Spatially confined assembly of monodisperse ruthenium nanoclusters in a hierarchically ordered carbon electrode for efficient hydrogen evolution. *Angew. Chem. Int. Ed.* **57**, 5848-5852 (2018).
11. Su, J. et al. Ruthenium-cobalt nanoalloys encapsulated in nitrogen-doped graphene as active electrocatalysts for producing hydrogen in alkaline media. *Nat. Commun.* **8**, 14969 (2017).
12. Jiang, P. et al. Pt-like electrocatalytic behavior of Ru-MoO_2 nanocomposites for the hydrogen evolution reaction. *J. Mater. Chem. A* **5**, 5475-5485 (2017).

13. Wang, J., Wei, Z., Mao, S., Li, H. & Wang, Y. Highly uniform Ru nanoparticles over N-doped carbon: pH and temperature-universal hydrogen release from water reduction. *Energy Environ. Sci.* **11**, 800-806 (2018).
14. Xing, Z., Han, C., Wang, D., Li, Q. & Yang, X. Ultrafine Pt nanoparticle-decorated $Co(OH)_2$ nanosheet arrays with enhanced catalytic activity towards hydrogen evolution. *ACS Catal.* **7**, 7131-7135 (2017).
15. Wang, P., Jiang, K., Wang, G., Yao, J. & Huang, X. Phase and interface engineering of platinum-nickel nanowires for efficient electrochemical hydrogen evolution. *Angew. Chem. Int. Ed.* **55**, 12859-12863 (2016).
16. Jiang, P. et al. Tuning the activity of carbon for electrocatalytic hydrogen evolution via an iridium-cobalt alloy core encapsulated in nitrogen-doped carbon cages. *Adv. Mater.* **30**, 1705324 (2018).
17. Cao, Z. et al. Platinum-nickel alloy excavated nano-multipods with hexagonal close-packed structure and superior activity towards hydrogen evolution reaction. *Nat. Commun.* **8**, 15131 (2017).
18. Zheng, Y. et al. High electrocatalytic hydrogen evolution activity of an anomalous ruthenium catalyst. *J. Am. Chem. Soc.* **138**, 16174-16181 (2016).
19. Yoon, D. et al. Cactus-like hollow $Cu_{2-x}S$@Ru nanoplates as excellent and robust electrocatalysts for the alkaline hydrogen evolution reaction. *Small* **13**, 1700052 (2017).
20. Wu, H. B., Xia, B. Y., Yu, L., Yu, X.-Y. & Lou, X. W. Porous molybdenum carbide nano-octahedrons synthesized via confined carburization in metal-organic frameworks for efficient hydrogen production. *Nat. Commun.* **6**, 6512 (2015).
21. Chen, P. et al. Phase-transformation engineering in cobalt diselenide realizing enhanced catalytic activity for hydrogen evolution in an alkaline medium. *Adv. Mater.* **28**, 7527-7532 (2016).
22. Ha, Y., Shi, L., Chen, Z. & Wu, R. Phase-transited lysozyme-driven formation of self-supported Co_3O_4@C nanomeshes for overall water splitting. *Adv. Sci.* 1900272 (2019).
23. Zhang, L. et al. Electrosynthesis of Co_3O_4 and $Co(OH)_2$ ultrathin nanosheet arrays for efficient electrocatalytic water splitting in alkaline and neutral media. *Nano Res.* **11**, 323-333 (2018).
24. Yu, F. et al. High-performance bifunctional porous non-noble metal phosphide catalyst for overall water splitting. *Nat. Commun.* **9**, 2551 (2018).

25. Li, H. et al. Systematic design of superaerophobic nanotube-array electrode comprised of transition-metal sulfides for overall water splitting. *Nat. Commun.* **9**, 2452 (2018).
26. Hui, L. et al. Overall water splitting by graphdiyne-exfoliated and-sandwiched layered double-hydroxide nanosheet arrays. *Nat. Commun.* **9**, 5309 (2018).
27. Li, H. et al. Amorphous nickel-cobalt complexes hybridized with 1T-phase molybdenum disulfide via hydrazine-induced phase transformation for water splitting. *Nat. Commun.* **8**, 15377 (2017).
28. Jia, Y. et al. A heterostructure coupling of exfoliated Ni-Fe hydroxide nanosheet and defective graphene as a bifunctional electrocatalyst for overall water splitting. *Adv. Mater.* **29**, 1700017 (2017).
29. Wu, Y. et al. Overall water splitting catalyzed efficiently by an ultrathin nanosheet-built, hollow, Ni_3S_2-based electrocatalyst. *Adv. Funct. Mater.* **26**, 4839-4847 (2016).
30. Liu, Y. et al. Coupling sub-nanometric copper cluster with quasi-amorphous cobalt sulfide yields efficient and robust electrocatalysts for water splitting reaction. *Adv. Mater.* **29**, 1606200 (2017).
31. Wang, H. et al. Bifunctional non-noble metal oxide nanoparticle electrocatalysts through lithium-induced conversion for overall water splitting. *Nat. Commun.* **6**, 7261 (2015).
32. Yu, L. et al. Cu nanowires shelled with NiFe layered double hydroxide nanosheets as bifunctional electrocatalysts for overall water splitting. *Energy Environ. Sci.* **10**, 1820-1827 (2017).
33. Yang, L. et al. Vertical growth of 2D amorphous $FePO_4$ nanosheet on Ni foam: outer and inner structure design for superior water splitting. *Adv. Mater.* **29**, 1704574 (2017).
34. Fan, X. et al. Defect-enriched iron fluoride-oxide nanoporous thin films bifunctional catalyst for water splitting. *Nat. Commun.* **9**, 1809 (2018).
35. Hu, C. et al. Synergism of geometric construction and electronic regulation: 3D Se-$(NiCo)S_x/(OH)_x$ nanosheets for highly efficient overall water splitting. *Adv. Mater.* **30**, 1705538 (2018).
